# Systematic analysis of nuclear gene function in respiratory growth and expression of the mitochondrial genome in *S. cerevisiae*

**DOI:** 10.15698/mic2020.09.729

**Published:** 2020-06-30

**Authors:** Maria Stenger, Duc Tung Le, Till Klecker, Benedikt Westermann

**Affiliations:** 1 Zellbiologie, Universität Bayreuth, 95440 Bayreuth, Germany.

**Keywords:** mitochondria, mitochondrial DNA, oxidative phosphorylation, petite mutant, yeast

## Abstract

The production of metabolic energy in form of ATP by oxidative phosphorylation depends on the coordinated action of hundreds of nuclear-encoded mitochondrial proteins and a handful of proteins encoded by the mitochondrial genome (mtDNA). We used the yeast *Saccharomyces cerevisiae* as a model system to systematically identify the genes contributing to this process. Integration of genome-wide high-throughput growth assays with previously published large data sets allowed us to define with high confidence a set of 254 nuclear genes that are indispensable for respiratory growth. Next, we induced loss of mtDNA in the yeast deletion collection by growth on ethidium bromide-containing medium and identified twelve genes that are essential for viability in the absence of mtDNA (i.e. *petite*-negative). Replenishment of mtDNA by cytoduction showed that respiratory-deficient phenotypes are highly variable in many yeast mutants. Using a mitochondrial genome carrying a selectable marker, *ARG8*^*m*^, we screened for mutants that are specifically defective in maintenance of mtDNA and mitochondrial protein synthesis. We found that up to 176 nuclear genes are required for expression of mitochondria-encoded proteins during fermentative growth. Taken together, our data provide a comprehensive picture of the molecular processes that are required for respiratory metabolism in a simple eukaryotic cell.

## INTRODUCTION

Most eukaryotic cells rely on mitochondrial respiration to liberate energy from metabolites and convert it to the universal energy currency, ATP. This process, also called oxidative phosphorylation, is performed by the respiratory chain. Large multisubunit protein complexes in the mitochondrial inner membrane transfer electrons from reduced substrates provided by the citric acid cycle to molecular oxygen. At the same time, they pump protons from the matrix across the inner membrane into the intermembrane space. This proton gradient fuels the ATP synthase that operates like a molecular turbine and uses the proton motive force for the synthesis of ATP [1, 2]. The respiratory chain complexes are mosaics of subunits encoded by nuclear and mitochondrial genes. Defects result in devastating diseases [3] and the accumulation of mutations in the mitochondrial genome is thought to constitute an important factor contributing to aging [4–6].

The mitochondrial proteome consists of about 900 (in yeast) to 1,500 (in humans) different proteins [7, 8]. While most of the mitochondrial proteins are encoded by nuclear genes and imported in a post-translational manner [9], only a handful of proteins are encoded by the mitochondrial DNA (mtDNA). The gene content of mitochondrial genomes varies between organisms. In general, mtDNAs encode the two mitochondrial rRNAs, a partial or full complement of tRNAs, some subunits of the respiratory chain complexes, and, at least in some organisms, some protein subunits of the mitochondrial ribosome [10]. In humans, the mtDNA encodes 13 respiratory chain subunits, including seven subunits of the NADH dehydrogenase (complex I), one subunit of the cytochrome *bc*_*1*_ complex (complex III), three subunits of the cytochrome *c* oxidase (complex IV), and two subunits of the ATP synthase (complex V) [4]. In budding yeast *Saccharomyces cerevisiae* the mtDNA encodes seven respiratory chain subunits and one ribosomal subunit [11]: the *COB* gene encodes cytochrome *b* (complex III), *COX1, COX2*, and *COX3* encode subunits of complex IV, *ATP6, ATP8*, and *ATP9* encode subunits of complex V, and *VAR1* encodes a protein of the small subunit of the mitochondrial ribosome. It is estimated that only about 15% of the mitochondrial proteins are directly involved in energy metabolism, while 20-25% of the mitochondrial proteome is required to maintain mtDNA and orchestrate mitochondrial gene expression [7].

*S. cerevisiae* is a powerful model organism to study the role of mitochondria in energy metabolism because it can satisfy its energy requirements by either fermentation or respiration, depending on the available carbon source. Oxidative phosphorylation and mtDNA are dispensable as long as yeast cells are grown on fermentable carbon sources, such as glucose, fructose, or galactose. Even in the presence of oxygen, glycolytic fermentation with ethanol and CO_2_ as end products is the preferred metabolic pathway for the generation of ATP. Most respiratory functions are repressed under these conditions (catabolite repression). Only when fermentable carbon sources become limiting, genes required for oxidative phosphorylation are induced and ATP is produced by metabolizing non-fermentable carbon sources, such as ethanol, glycerol, or lactate [12, 13]. Respiratory-deficient yeast mutants are termed *petite* (French: small) or *pet* because they form small colonies on non-fermentable media with limiting amounts of fermentable carbon sources [14, 15]. Cytoplasmic *petite* mutants contain mutations or lesions in the mitochondrial genome [*rho*^-^] or completely lack mtDNA [*rho*^*0*^] whereas nuclear *petite* mutants contain mutations of genes located in the nuclear genome. Nuclear *pet* genes encode enzymes of the citric acid cycle, subunits and assembly factors of the respiratory chain, proteins involved in maintenance and inheritance of mtDNA, factors required for mitochondrial transcription and translation, and proteins involved in mitochondrial dynamics and other functions [16–18]. In contrast to most other eukaryotes, including many yeasts, *S. cerevisiae* does not have a respiratory chain complex I [19, 20]. Its function has been replaced by an alternative, single amino acid chain NADH dehydrogenase, Ndi1, which transfers electrons from NADH to ubiquinone but does not pump protons [21, 22].

Several large-scale studies contributed to the identification of nuclear *pet* genes in yeast [18, 23–26]. However, these studies yielded largely different results, and a consensus of the complement of genes required for respiratory growth and maintenance of the mitochondrial genome in yeast is still lacking. We developed an integrative experimental approach for genome-wide mutant analysis, generating a high confidence set of nuclear *pet* genes and a comprehensive list of genes required for maintenance of mtDNA. We are confident that our data will be a valuable resource to estimate the contribution of particular genes and cellular pathways to respiratory growth and expression of mitochondria-encoded proteins in budding yeast.

## RESULTS AND DISCUSSION

### Definition of a high confidence set of nuclear *pet* genes

The yeast deletion collection contains mutants of all ca. 4,800 non-essential yeast genes (corresponding to about 83% of all yeast genes) and constitutes a great resource for the systematic and genome-wide analysis of gene function [27–29]. Three previous studies have made use of it to identify *pet* genes by plating the strains on media containing non-fermentable carbon sources: Dimmer *et al.* identified 341 *pet* mutants in the homozygous diploid collection [23], Luban *et al.* identified 355 *pet* mutants in the *MAT*a collection [25], and Merz and Westermann identified 319 *pet* mutants in the *MAT*α collection [18]. Even though the numbers of *pet* genes identified in each individual screen were very similar, the overlap was surprisingly small: only 176 *pet* mutants were found in all of the three screens [18].

Which factors may account for largely diverging results in three very similar screens? The complement of genes covered by different versions of the yeast deletion collection may vary. For example, deletion mutants of 140 newly identified small ORFs became available only in 2006 [30], i.e. after two of the three screens were performed. Also, there are several errors associated with the strains of the deletion collections [28]. Phenotypes caused by second-site mutations that segregate away from the marker allele were estimated to occur in about 6.5% of haploid deletion mutants [31]. Up to 8% of the deletion strains are thought to have retained a wild type copy of the targeted gene presumably because of aneuploidy or a duplication event [31–33]. Deletions of ORFs may result in compensatory second-site mutations, often affecting nutrient responses and/or heat stress-induced cell death [34]. Deletions may also affect the function of neighboring genes [35]. Last but not least, good yeast husbandry is required to minimize errors associated with the deletion collection [28]. It has been estimated that over one third of the data obtained from a primary screen of the collection may consist of false positives or false negatives [32].

**Figure 1:**
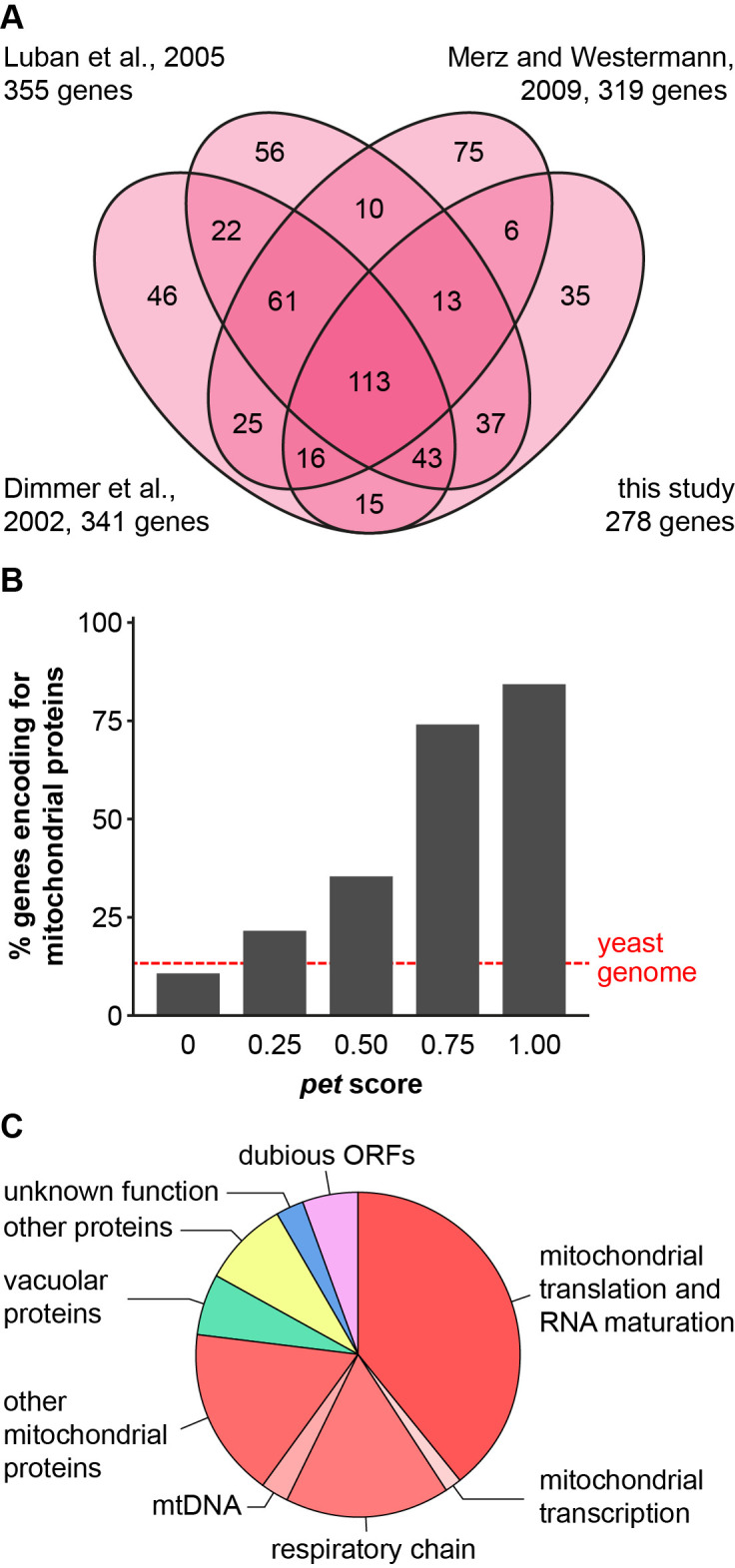
FIGURE 1: Defining a set of high confidence nuclear *pet* mutants. **(A)** Venn diagram comparing the results of four different screens ([18, 23, 25] and this study) for mutants with a *pet* phenotype. In each case, genome-wide collections of viable deletion mutants were analyzed and a *pet* phenotype was attributed to strains that were unable to grow on rich media containing glycerol as nonfermentable carbon source. **(B)** A *pet* score was derived from the four screens depicted in A by comparing the times a *pet* phenotype was reported for each gene to the times the deletion mutant was analyzed. See text for details. Viable deletion mutants were grouped according to their *pet* score and analyzed for the percentage of encoded proteins that were found in a high confidence mitochondrial proteome [8]. A detailed list containing the results from A and B can be found in Table S1. **(C)** Mutants with a *pet* score higher than 0.5, referred to as high confidence *pet* mutants, were manually grouped into functional categories. Lists of the genes present in each group can be found in Tables 1 and S2.

To define a high confidence set of nuclear *pet* genes in *S. cerevisiae*, we first scored the growth of the *MAT*a deletion collection on plates with non-fermentable carbon sources. Using a high precision pinning robot, cells were first plated in a high density array on glucose-containing YPD plates, grown to colonies, and then transferred to glycerol-containing YPG plates. We observed that 278 deletion mutants failed to grow on YPG. When we compared this new list of *pet* genes to the previous three screens we found 113 mutants that were respiratory-deficient in all four screens (**Figure 1A**).

We then calculated a *pet* score for all yeast genes. We defined the *pet* score as the number of times a gene was identified as a *pet* gene divided by the number of times this deletion mutant was screened; i.e. the maximum *pet* score of 1 means that a deletion mutant was always found to be respiratory-deficient, whereas a *pet* score of 0.25 means that a mutant was found to be respiratory-deficient in only one out of four screens. These results are compiled in Table S1. This table contains the systematic gene name, the standard gene name, a brief description of the protein function according to the Saccharomyces Genome Database, SGD [36], the presence of the protein in a high confidence mitochondrial proteome [8], the results of the four *pet* screens ([18, 23, 25] and this work), and the *pet* score.

Next, we correlated the *pet* score with a known localization of the gene product in mitochondria. We found that about 84% of the genes with a *pet* score of 1 and about 74% of the genes with a *pet* score of 0.75 encode mitochondrial proteins. This fraction was reduced to about 35% for a *pet* score of 0.5 and about 22% for a *pet* score of 0.25 (**Figure 1B**). Thus, a high *pet* score clearly correlates with a mitochondrial function of the gene product. We propose that genes with a *pet* score higher than 0.5 should be regarded as high confidence *pet* genes. This definition requires that a high confidence *pet* mutant has to repeatedly show a respiratory-deficient phenotype, but it does not exclude mutants that yielded one false-negative result. According to this definition there are 254 high confidence *pet* genes in yeast, 79% of which encode mitochondrial proteins (**Tables 1** and S2).

**TABLE 1.** **High confidence *pet* genes in yeast.** Genes were manually grouped into the functional categories shown in Figure 1C. More details can be found in Tables S1 and S2.

**Standard name**	**ORF**	**Standard name**	**ORF**	**Standard name**	**ORF**
**Mitochondrial translation and RNA maturation**					
*AEP1*	*YMR064W*	*MRPL15*	*YLR312W-A*	*MSK1*	*YNL073W*
*AEP2*	*YMR282C*	*MRPL16*	*YBL038W*	*MSM1*	*YGR171C*
*AEP3*	*YPL005W*	*MRPL17*	*YNL252C*	*MSR1*	*YHR091C*
*AIM10*	*YER087W*	*MRPL19*	*YNL185C*	*MSS51*	*YLR203C*
*ATP22*	*YDR350C*	*MRPL20*	*YKR085C*	*MST1*	*YKL194C*
*ATP25*	*YMR098C*	*MRPL22*	*YNL177C*	*MSW1*	*YDR268W*
*CBP1*	*YJL209W*	*MRPL23*	*YOR150W*	*MSY1*	*YPL097W*
*CBP2*	*YHL038C*	*MRPL24*	*YMR193W*	*MTG1*	*YMR097C*
*CBS1*	*YDL069C*	*MRPL25*	*YGR076C*	*MTG2*	*YHR168W*
*CBS2*	*YDR197W*	*MRPL27*	*YBR282W*	*NAM2*	*YLR382C*
*CCM1*	*YGR150C*	*MRPL31*	*YKL138C*	*PET111*	*YMR257C*
*DIA4*	*YHR011W*	*MRPL32*	*YCR003W*	*PET112*	*YBL080C*
*GEP3*	*YOR205C*	*MRPL33*	*YMR286W*	*PET122*	*YER153C*
*GEP5*	*YLR091W*	*MRPL36*	*YBR122C*	*PET123*	*YOR158W*
*HER2*	*YMR293C*	*MRPL37*	*YBR268W*	*PET309*	*YLR067C*
*IFM1*	*YOL023W*	*MRPL38*	*YKL170W*	*PET494*	*YNR045W*
*IMG1*	*YCR046C*	*MRPL4*	*YLR439W*	*PET54*	*YGR222W*
*IMG2*	*YCR071C*	*MRPL40*	*YPL173W*	*RMD9*	*YGL107C*
*MEF1*	*YLR069C*	*MRPL49*	*YJL096W*	*RML2*	*YEL050C*
*MEF2*	*YJL102W*	*MRPL51*	*YPR100W*	*RRF1*	*YHR038W*
*MHR1*	*YDR296W*	*MRPL6*	*YHR147C*	*RRG8*	*YPR116W*
*MNE1*	*YOR350C*	*MRPL7*	*YDR237W*	*RSM18*	*YER050C*
*MRF1*	*YGL143C*	*MRPL8*	*YJL063C*	*RSM19*	*YNR037C*
*MRP1*	*YDR347W*	*MRPL9*	*YGR220C*	*RSM22*	*YKL155C*
*MRP10*	*YDL045W-A*	*MRPS12*	*YNR036C*	*RSM23*	*YGL129C*
*MRP17*	*YKL003C*	*MRPS16*	*YPL013C*	*RSM24*	*YDR175C*
*MRP20*	*YDR405W*	*MRPS28*	*YDR337W*	*RSM27*	*YGR215W*
*MRP21*	*YBL090W*	*MRPS5*	*YBR251W*	*RSM7*	*YJR113C*
*MRP4*	*YHL004W*	*MRPS8*	*YMR158W*	*SLM5*	*YCR024C*
*MRP51*	*YPL118W*	*MRS1*	*YIR021W*	*SLS1*	*YLR139C*
*MRP7*	*YNL005C*	*MRX14*	*YDR115W*	*SWS2*	*YNL081C*
*MRPL10*	*YNL284C*	*MSD1*	*YPL104W*	*TUF1*	*YOR187W*
*MRPL11*	*YDL202W*	*MSE1*	*YOL033W*		
*MRPL13*	*YKR006C*	*MSF1*	*YPR047W*		
**Mitochondrial transcription**					
*MSS116*	*YDR194C*	*MTF2*	*YDL044C*		
*MTF1*	*YMR228W*	*RPO41*	*YFL036W*		
**Respiratory chain components and assembly factors**					
*ATP1*	*YBL099W*	*COR1*	*YBL045C*	*IMP1*	*YMR150C*
*ATP10*	*YLR393W*	*COX10*	*YPL172C*	*IMP2*	*YMR035W*
*ATP11*	*YNL315C*	*COX11*	*YPL132W*	*MSS2*	*YDL107W*
*ATP12*	*YJL180C*	*COX12*	*YLR038C*	*PET100*	*YDR079W*
*ATP15*	*YPL271W*	*COX18*	*YGR062C*	*PET117*	*YER058W*
*ATP17*	*YDR377W*	*COX19*	*YLL018C-A*	*QCR2*	*YPR191W*
*ATP2*	*YJR121W*	*COX20*	*YDR231C*	*QCR7*	*YDR529C*
*ATP3*	*YBR039W*	*COX5A*	*YNL052W*	*QCR8*	*YJL166W*
*ATP4*	*YPL078C*	*COX6*	*YHR051W*	*RIP1*	*YEL024W*
*ATP5*	*YDR298C*	*COX7*	*YMR256C*	*SCO1*	*YBR037C*
*ATP7*	*YKL016C*	*COX9*	*YDL067C*	*SDH1*	*YKL148C*
*BCS1*	*YDR375C*	*CYC3*	*YAL039C*	*SDH2*	*YLL041C*
*CBP3*	*YPL215W*	*CYT1*	*YOR065W*	*SDH5*	*YOL071W*
*CBP4*	*YGR174C*	*CYT2*	*YKL087C*	*SHY1*	*YGR112W*
**mtDNA**					
*ABF2*	*YMR072W*	*MIP1*	*YOR330C*	*RIM1*	*YCR028C-A*
*HMI1*	*YOL095C*	*MSH1*	*YHR120W*		
*MGM101*	*YJR144W*	*PIF1*	*YML061C*		
**Other mitochondrial proteins**					
*ACO1*	*YLR304C*	*FTR1*	*YER145C*	*MDJ1*	*YFL016C*
*AFG3*	*YER017C*	*FUM1*	*YPL262W*	*MET7*	*YOR241W*
*AIM22*	*YJL046W*	*FZO1*	*YBR179C*	*MGM1*	*YOR211C*
*CAT5*	*YOR125C*	*GCV3*	*YAL044C*	*NFU1*	*YKL040C*
*CEM1*	*YER061C*	*GGC1*	*YDL198C*	*OAR1*	*YKL055C*
*COQ1*	*YBR003W*	*GRX5*	*YPL059W*	*OCT1*	*YKL134C*
*COQ10*	*YOL008W*	*HEM14*	*YER014W*	*OXA1*	*YER154W*
*COQ2*	*YNR041C*	*HTD2*	*YHR067W*	*PPA2*	*YMR267W*
*COQ3*	*YOL096C*	*IBA57*	*YJR122W*	*PPT2*	*YPL148C*
*COQ4*	*YDR204W*	*ISA1*	*YLL027W*	*SOM1*	*YEL059C-A*
*COQ5*	*YML110C*	*ISA2*	*YPR067W*	*SSQ1*	*YLR369W*
*COQ6*	*YGR255C*	*KGD2*	*YDR148C*	*SUV3*	*YPL029W*
*COQ9*	*YLR201C*	*LIP2*	*YLR239C*	*YTA12*	*YMR089C*
*DSS1*	*YMR287C*	*LPD1*	*YFL018C*		
*ETR1*	*YBR026C*	*MCT1*	*YOR221C*		
**Vacuolar proteins**					
*DID4*	*YKL002W*	*VMA21*	*YGR105W*	*VMA6*	*YLR447C*
*VMA1*	*YDL185W*	*VMA22*	*YHR060W*	*VMA8*	*YEL051W*
*VMA10*	*YHR039C-A*	*VMA3*	*YEL027W*	*VMA9*	*YCL005W-A*
*VMA11*	*YPL234C*	*VMA4*	*YOR332W*	*VPS16*	*YPL045W*
*VMA16*	*YHR026W*	*VMA5*	*YKL080W*	*VPS33*	*YLR396C*
**Other proteins**					
*AFT1*	*YGL071W*	*HAP2*	*YGL237C*	*RPL1B*	*YGL135W*
*BUD25*	*YER014C-A*	*HAP3*	*YBL021C*	*SNF1*	*YDR477W*
*CTR1*	*YPR124W*	*HAP4*	*YKL109W*	*SNF4*	*YGL115W*
*CYS3*	*YAL012W*	*HAP5*	*YOR358W*	*SWI3*	*YJL176C*
*DEF1*	*YKL054C*	*LCB5*	*YLR260W*	*TPD3*	*YAL016W*
*DOC1*	*YGL240W*	*MAC1*	*YMR021C*	*VPS34*	*YLR240W*
*FBP1*	*YLR377C*	*RNR4*	*YGR180C*		
*GRR1*	*YJR090C*	*RPB9*	*YGL070C*		
**Proteins of unknown function**					
*IRC19*	*YLL033W*	*RRG9*	*YNL213C*	*YNL184C*	*YNL184C*
*RRG1*	*YDR065W*	*SOV1*	*YMR066W*		
*RRG7*	*YOR305W*	*YDR114C*	*YDR114C*		
**Dubious ORFs**					
*YBL100C*	*YBL100C*	*YGR219W*	*YGR219W*	*YNL170W*	*YNL170W*
*YCL007C*	*YCL007C*	*YJL120W*	*YJL120W*	*YOR200W*	*YOR200W*
*YDL068W*	*YDL068W*	*YJR114W*	*YJR114W*	*YOR331C*	*YOR331C*
*YDR230W*	*YDR230W*	*YKL169C*	*YKL169C*	*YPR099C*	*YPR099C*
*YGL218W*	*YGL218W*	*YLR202C*	*YLR202C*		

High confidence *pet* genes encode proteins involved in mitochondrial translation and RNA maturation (39.4%), respiratory chain components and assembly factors (16.5%), mitochondrial transcription (1.6%), mtDNA metabolism (2.8%), other mitochondrial proteins (16.9%), vacuolar proteins (5.9%), other known proteins (8.7%), proteins of unknown function (2.8%), and dubious ORFs (5.5%) (**Figure 1C**, **Tables 1** and S2). All dubious ORFs overlap with genes with known functions in mitochondria (Table S2).

A large number of mutants lacking subunits or assembly factors of the vacuolar ATPase (vATPase) were found to have a high *pet* score, including Δ*vma1*, Δ*vma3*, Δ*vma4*, Δ*vma5*, Δ*vma6*, Δ*vma8*, Δ*vma9*, Δ*vma10*, Δ*vma11*, Δ*vma16*, Δ*vma21*, and Δ*vma22*. This is in accordance with previously published observations (see e.g. [18, 37–39]). Addition of iron or copper to the medium restores respiratory growth of vATPase mutants [38, 39], indicating that they retained respiratory competence. In line with this, we found that several mutants lacking proteins involved in metal ion homeostasis show a high *pet* score (Table S1). These include the high affinity plasma membrane iron or copper transporters Ftr1 and Ctr1 and the iron or copper homeostasis transcription factors Aft1 and Mac1. Intriguingly, mitochondrial iron content and biogenesis of iron sulfur cluster proteins are reduced by loss of vATPase activity, and supply of exogenous iron rescues this defect [40–42]. Furthermore, defects in vacuole function result in elevated cytosolic cysteine, which in turn impairs mitochondrial respiration by limiting iron availability [42]. Taken together, it appears that the *pet* phenotype of vATPase mutants is caused by compromised metal ion homeostasis in the cytosol.

It should be noted that *pet* phenotypes may vary between different strain backgrounds. Our analysis is based on genome-wide screens with the yeast deletion collection that was generated using strains BY4741, BY4742, BY4743, and to a lesser extent BY4730 and BY4739 [29]. Strains of the BY series [43] are derivatives of the widely used laboratory strain S288C [44], which also was used as a source for sequencing of the yeast genome [45]. Quantitative trait locus (QTL) mapping revealed alleles of four genes that affect respiratory growth of BY strains in comparison to other laboratory strains and wild type isolates [46]: *MKT1-30D*, encoding a putative translation regulator that translocates to P bodies upon ethanol stress [47]; *sal1-1*, encoding an ATP/ADP carrier in the mitochondrial inner membrane [48]; *CAT5-91I*, encoding a mitochondrial protein required for ubiquinone (coenzyme Q) biosynthesis [49]; and *MIP1-661A*, encoding the mitochondrial DNA polymerase [50]. Furthermore, S288C-derived strains carry a defective *Ty1* transposon inserted in the 3' region of the *HAP1* ORF. This insertion severely compromises the function of the Hap1 transcription factor which is involved in the regulation of gene expression in response to levels of heme and oxygen, including respiratory chain components [51]. Taken together, these alleles make the strains that were used to construct the deletion collection particularly vulnerable to perturbations of mitochondrial functions.

### *pet* phenotypes are highly variable for many gene deletions

*Petite* phenotypes may be highly variable even within the same genetic background. Strikingly, deletions of the dubious ORFs Δ*ygl218w* and Δ*ynl170w* have high *pet* scores (0.75), whereas deletions of their overlapping protein-coding genes Δ*mdm34* and Δ*psd1*, respectively, have *pet* scores of 0 (see Table S2). A detailed study on Mdm34 (alternative name Mmm2) reported that Δ*mdm34* cells were initially deficient in growth on plates containing non-fermentable carbon sources, but single colonies were clearly visible after 10-14 days and could immediately grow after transfer to fresh medium [52]. Another study showed that Δ*mdm34* mutants rapidly accumulate suppressor mutations in the *VPS13* gene [53]. Similarly, the Δ*psd1* mutant was found to have a strong growth defect on YPG but only a moderate growth defect on YPEG plates [54]. Furthermore, a previous study showed that the Δ*ygl218w* mutant had lost its mtDNA in the original yeast deletion library but was able to stably maintain it after cytoduction, and Δ*ynl170w* belonged to a group of 77 mutants that grew on non-fermentable carbon sources only after cells had the chance to adapt to the medium [18]. Thus, Δ*mdm34*, Δ*ygl218w*, Δ*psd1*, and Δ*ynl170w* – and certainly many other deletion strains – show highly variable growth behaviors on non-fermentable carbon sources.

**Figure 2:**
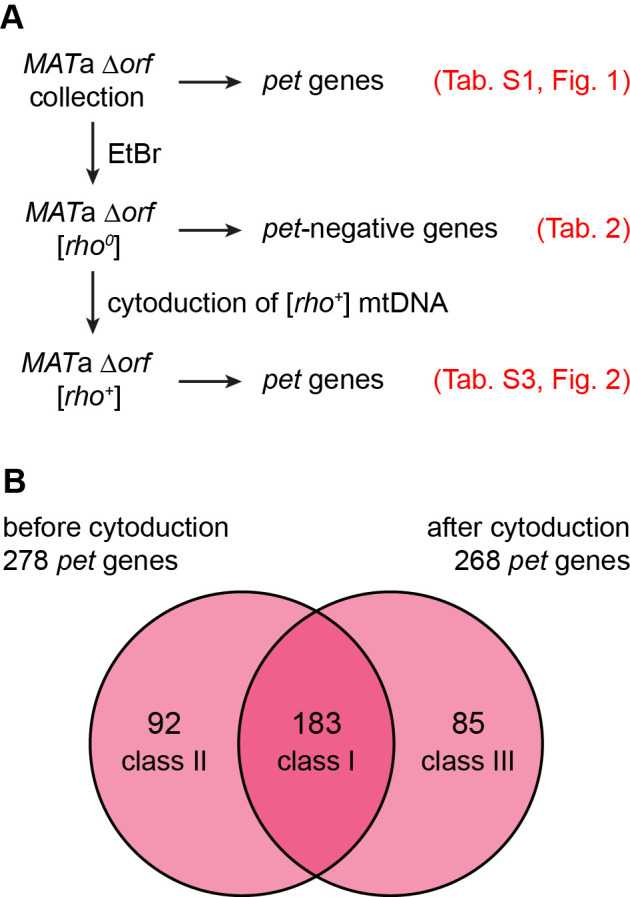
FIGURE 2: Contribution of mtDNA maintenance to the variability of *pet* phenotypes. **(A)** Flow chart depicting the experimental outline. In brief, the whole deletion collection was treated with EtBr to induce loss of mtDNA. Functional [*rho*^*+*^] mtDNA was re-introduced into each strain by cytoduction. The resulting [*rho*^*+*^] deletion collection was tested for growth on rich media containing glycerol as nonfermentable carbon source. See text for details. **(B)** Venn diagram comparing the sets of deletion mutants that showed a *pet* phenotype before and after EtBr-treatment and cytoduction. The mutants were grouped into three classes: Mutants that exhibited a *pet* phenotype before and after cytoduction were classified as class I mutants, those that were rescued by cytoduction were grouped into class II, and mutants that were unable to grow on media with glycerol as the carbon source only after cytoduction are referred to as class III mutants. Three of the 278 *pet* mutants from the original deletion collection exhibited a *petite*-negative phenotype and were omitted from the analysis.

Mitochondrial genome instability is responsible for the occurrence of spontaneous *petite* mutants in populations of yeast cells. While true wild type and domesticated *S. cerevisiae* strains give rise to relatively few [*rho*^-^] or [*rho*^*0*^] colonies, many laboratory strains produce high frequencies of spontaneous *petites* [55]. The *sal1-1, CAT5-91I*, and *MIP1-661A* alleles present in the strains of the BY series are responsible for an about 100-fold elevated rate of loss of mtDNA [46]. We reasoned that a significant number of mutants may have shown a *pet* phenotype in screens of the yeast deletion collection because of spontaneous loss of mtDNA. To test this, we first induced loss of mtDNA by plating the *MAT*a deletion collection on ethidium bromide (EtBr) containing medium [56] to avoid heteroplasmy. We then freshly introduced wild type mtDNA by cytoduction [57] and assayed again growth on non-fermentable carbon sources (**Fig. 2A**).

The *MAT*a deletion collection was passaged three times on glucose-containing YPD medium supplemented with EtBr to induce loss of mtDNA. Successful elimination of the mitochondrial genome was verified by lack of growth of all strains on glycerol-containing YPG medium and absence of mtDNA nucleoids in 24 randomly chosen mutants upon DAPI staining and fluorescence microscopy. Twelve strains failed to grow on EtBr-containing medium (**Table 2**). This group of mutants is expected to include *petite*-negative mutants, i.e. mutants that cannot live without their mtDNA, even when they are grown on fermentable carbon sources. In the absence of a functional respiratory chain the F_1_-ATP synthase and the major ADP/ATP carrier, Pet9, are required to maintain a membrane potential, Δψ, across the inner membrane. Under these conditions, the F_1_-ATP synthase hydrolyzes ATP, generating excess ADP in the matrix. ADP^3-^ is then exchanged for ATP^4-^ imported from the cytosol by ADP/ATP carriers in the inner membrane. The import of one net negative charge per pair of transported adenine nucleotides builds up a membrane potential, which is required to maintain protein import into the matrix, an activity which is essential for cell viability [58, 59]. This explains why mutants Δ*atp1*, lacking the alpha subunit of the F_1_-ATP synthase, and Δ*atp11* and Δ*atp12*, lacking F_1_-ATP synthase assembly factors, were unable to grow on EtBr-containing medium. Δ*tim18*, lacking a subunit of the TIM22 protein import complex in the inner membrane, was already identified in a previous screen for *petite*-negative mutants [59]. We have not verified a role of the other genes in conferring a *petite*-negative phenotype.

**TABLE 2.** **Mutants that were unable to grow on EtBr-containing medium.** Descriptions of gene functions were taken from the Saccharomyces Genome Database [36] with some manual annotations.

**Standard name**	**ORF**	**Gene function**
*APD1*	*YBR151w*	Protein of unknown function
*ATP1*	*YBL099w*	Alpha subunit of the F_1_ sector of mitochondrial F_1_F_o_ ATP synthase
*ATP11*	*YNL315c*	Molecular chaperone required for the assembly of alpha and beta subunits into the F_1_ sector of mitochondrial F_1_F_o_ ATP synthase
*ATP12*	*YJL180c*	Assembly factor for F_1_ sector of mitochondrial F_1_F_o_ ATP synthase
*CLC1*	*YGR167w*	Clathrin light chain
*NAT3*	*YPR131c*	Catalytic subunit of the NatB N-terminal acetyltransferase
*OCH1*	*YGL038c*	Mannosyltransferase of the cis-Golgi apparatus
*ROX3*	*YBL093c*	Subunit of the RNA polymerase II mediator complex
*RPB9*	*YGL070c*	RNA polymerase II subunit B12.6
*SSD1*	*YDR293c*	Translational repressor
*SWI6*	*YLR182w*	Transcription cofactor
*TIM18*	*YOR297c*	Component of the mitochondrial TIM22 complex, involved in insertion of polytopic proteins into the inner membrane

Previous work by Dunn *et al.* [59] reported the identification of twelve *petite*-negative mutants in a screen of 3,791 haploid yeast deletion strains. It should be noted that the overlap between this screen an ours is surprisingly small (Δ*tim18* is the only mutant that was identified both by Dunn *et al.* and in the present study). While the role of the F_1_ ATP synthase in conferring a *petite* negative phenotype is well established [58], the Δ*atp2* mutant was not detected in our screen, and Δ*atp1*, Δ*atp11*, and Δ*atp12* were not found by Dunn *et al.* We consider it likely that the *petite*-negative phenotype is highly variable, like the *pet* phenotype, and its appearance might depend on strain background and experimental conditions.

In a next step we mated the *MAT*a [*rho*^*0*^] deletion collection with the cytoduction donor strain J1362, which is karyogamy-defective due to the *kar1*Δ*15* allele and carries a *Kluyveromyces lactis URA3* gene on each chromosome to allow counterselection on medium containing 5'FOA (5-fluoroorotic acid) [60]. After cytoduction and growth on 5'FOA we observed that the wild type mtDNA donated by J1362 restored the ability to grow on medium with non-fermentable carbon source, YPG, for most strains. 268 mutants failed to grow on YPG (Table S3). A comparison of this set of *pet* mutants with the set of 275 *pet* mutants that were originally present in this collection (i.e. 278 *pet* mutants minus three *petite*-negative *pet* mutants; listed in **Tables 2** and S1) revealed an overlap of 183 *pet* genes (**Fig. 2B**). Of these, 161 mutants, corresponding to 88%, are high confidence *pet* mutants.

92 mutants that were respiratory-deficient in the original collection were cured by cytoduction. Of these, only 29.3% are high confidence *pet* genes suggesting that they acquire a respiratory-deficient phenotype only under some circumstances. As the nuclear genome was not altered during the cytoduction experiment, we assume that second-site mutations, aneuploidy, or gene duplications in most cases are not responsible for the variations of growth behavior before and after cytoduction. It is reasonable to assume that at least some of the 92 mutants that were cured by cytoduction had lost their mtDNA after creation of the yeast deletion collection. For 41 mutants, some residual growth could be observed upon transfer to fresh YPG medium, indicating that functional mtDNA was present. To test whether the remaining 51 strains had lost their mitochondrial genome, we mated them with the Δ*mip1* mutant, which is [*rho*^*0*^]. We observed that 48 heterozygous diploid strains failed to grow on YPG, indicating that the parental strains indeed lacked functional mtDNA (Table S4). Thus, spontaneous loss of mtDNA contributes to the variations of *pet* phenotypes that are observed in a substantial number of yeast deletion mutants.

Unexpectedly, 85 mutants were respiratory-competent before growth on EtBr-containing medium and failed to be rescued by cytoduction. This group contains only nine high confidence *pet* mutants, corresponding to 10.6%, suggesting that most of these genes are dispensable for respiration. To test whether these are random effects, we repeated the cytoduction experiment and obtained 232 mutants that were respiratory-deficient after cytoduction. 213 of these mutants were already found in the first cytoduction experiment, indicating that the results are highly reproducible (Table S3). Of these, 46 respiratory-competent strains reproducibly became respiratory-deficient only after cytoduction. This relatively large number suggests that this effect is not random. However, it is unknown whether cytoduction is inefficient in these mutants, e.g. because of mating defects, or whether they acquired respiratory deficiency for other reasons. For example, it is possible that EtBr treatment induced second site mutations, as EtBr was shown to increase the mutation frequency of the nuclear genome [61].

Taken together, our results suggest that *pet* phenotypes of many gene deletions are highly variable, at least in BY laboratory yeast strains. It has been suggested that environmental factors, nutrient supply, and epigenetic mechanisms may contribute to the plasticity of *pet* phenotypes [18]. Thus, loss of mtDNA is only one of several reasons for an acquired *pet* phenotype. Alternative reasons may include an insufficient relief of catabolite repression, or the accumulation of irreversible damage independent of the presence of mtDNA.

### Genes required for expression and maintenance of the mitochondrial genome

**Figure 3:**
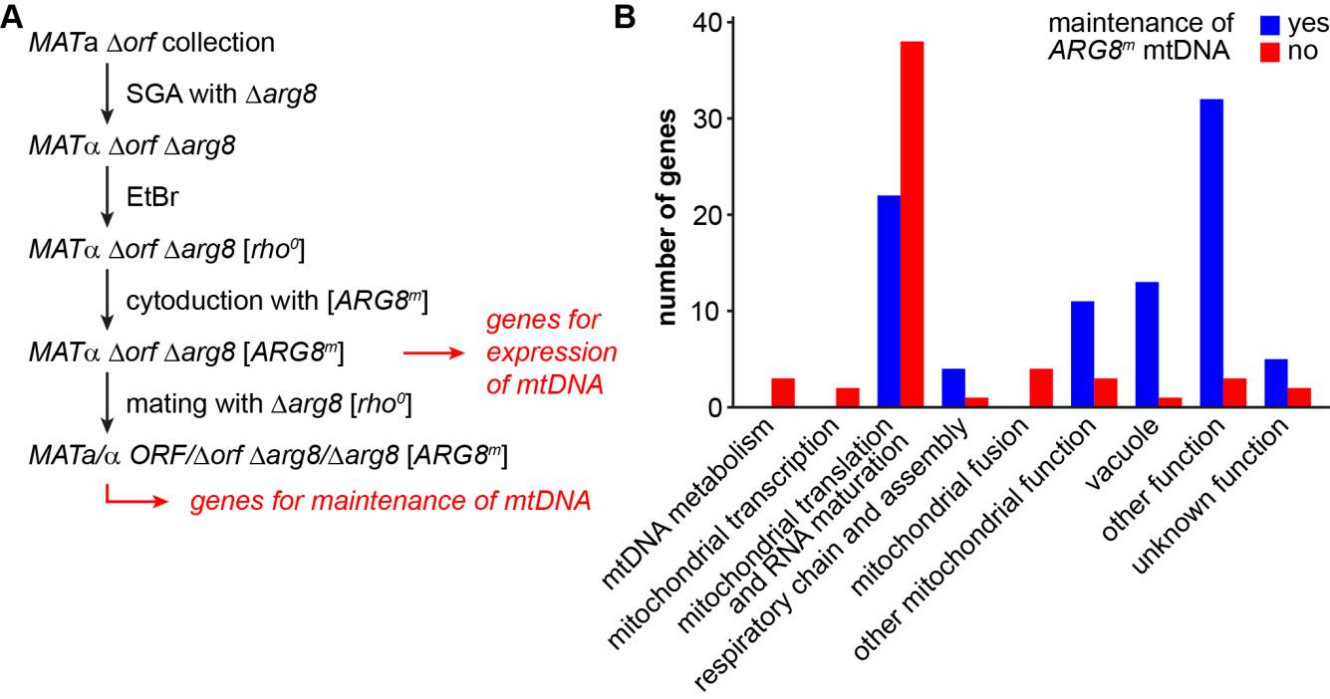
FIGURE 3: Defining the genes required for expression and maintenance of the mitochondrial genome. **(A)** Flow chart depicting the outline of the experiment. A Δ*orf* Δ*arg8* double mutant collection was generated using SGA technology (see methods). The mitochondrial genome was eliminated from all double mutants by treatment with EtBr. A functional mtDNA containing the *ARG8*^*m*^ allele was introduced into all strains by cytoduction. Mutants that were unable to grow on media lacking arginine after cytoduction were considered to have lost their mtDNA or to be unable to express the *ARG8*^*m*^ gene. To test for this, the resulting Δ*orf* Δ*arg8* [*ARG8*^*m*^] double mutant collection was crossed with a Δ*arg8* [*rho*^*0*^] strain and the resulting diploid strains were scored for growth on media lacking arginine. Mutants that were unable to grow were considered to suffer from mtDNA instability. See text for details. **(B)** The mutants that had lost their mtDNA or that were unable to express the *ARG8*^*m*^ gene were manually grouped into functional categories. Depicted is how often each functional group is represented among these two sets of mutants. Blue bars represent mutants that maintained the [*ARG8*^*m*^] mitochondrial genome, but were unable to express Arg8^m^ (i.e. the genes listed in Table 3; these are the “genes for expression of mtDNA” minus “genes for maintenance of mtDNA” in panel A). Red bars represent mutants that lost the [*ARG8*^*m*^] mitochondrial genome (i.e. the genes listed in Table 4; these are the “genes for maintenance of mtDNA” in panel A that could be confirmed by DAPI staining).

It is estimated that as many as 250 proteins might be required for the expression of only eight proteins that are encoded by the mitochondrial genome in yeast [62, 63]. To identify the genes that are specifically involved in this process, we devised a screen that allowed us to select for mitochondrial gene expression independent of respiratory functions and the carbon source of the medium (**Fig. 3A**). Arg8 is a nuclear-encoded mitochondrial enzyme that catalyzes the fourth step in the biosynthesis of the arginine precursor ornithine [64, 65]. The *ARG8*^*m*^ allele is a synthetic gene adapted to the mitochondrial genetic code and integrated into mtDNA. It fully complements a nuclear Δ*arg8* deletion at the level of cell growth and therefore can serve as an auxotrophic marker synthesized in mitochondria [66].

To screen for mutants defective in *ARG8*^*m*^ expression, we first introduced the genomic Δ*arg8* allele into the *MAT*a yeast deletion collection by synthetic genetic array (SGA) technology [67, 68] and obtained 4,523 mutants that were unable to grow in the absence of arginine (i.e. they are arginine-auxotroph). Then, we passaged the strains on EtBr-containing medium to induce loss of mtDNA, introduced a [*ARG8*^*m*^ *rho*^*+*^] mitochondrial genome [69] by cytoduction and scored for restoration of growth on glucose-containing minimal medium without arginine (i.e. arginine prototrophy). We obtained 198 mutants that were unable to grow on minimal medium lacking arginine (Table S5). This group of mutants is expected to pertain all nuclear genes that are essential for maintenance of mtDNA and expression of mitochondria-encoded proteins. Several deletion mutants were respiratory-competent, as judged by their *pet* score, but unable to grow on medium lacking arginine after cytoduction. This latter group includes mutants defective in certain steps of amino acid biosynthesis, including Δ*arg1*, Δ*arg3*, Δ*arg4*, Δ*arg5,6*, Δ*cpa1*, and Δ*cpa2*. It is conceivable that these strains are arginine-auxotroph because steps of amino acids metabolism other than Arg8-dependent synthesis of ornithine are affected. Furthermore, several mutants lack proteins known to be required during mating, including Δ*aga2*, Δ*erg6*, Δ*htl1*, Δ*sst2*, Δ*ste3*, and Δ*ste20.* We assume that these mutants remained arginine-auxotroph because they failed to receive the [*ARG8*^*m*^] mitochondrial genome by cytoduction. We excluded both groups and all dubious ORFs from further analysis, yielding a total number of 176 mutants that could not support mitochondrial protein synthesis. 97 (55%) of these mutants were high confidence *pet* mutants and 61% of the encoded proteins have a known mitochondrial localization.

The analysis of arginine auxotrophy of Δ*arg8* [*ARG8*^*m*^] yeast deletion strains allowed the identification of a set of genes required for expression of mitochondria-encoded proteins. However, it does not reveal whether the defect is at the level of maintenance of mtDNA or mitochondrial protein synthesis. To discriminate between these possibilities, we tested whether arginine prototrophy of the yeast deletion strains can be restored by mating with a Δ*arg8* [*rho*^*0*^] strain. We reasoned that growth on minimal media lacking arginine should be restored in the resulting heterozygous strain when the yeast deletion mutant contributes the *ARG8*^*m*^ gene on its mtDNA and the mating partner contributes the missing ORF required for Arg8^m^ expression. If, however, the yeast deletion strain is unable to maintain mtDNA the heterozygous strain will remain arginine-auxotroph because it lacks the *ARG8*^*m*^ allele.

We observed that mating restored arginine prototrophy in 87 deletion mutants, indicating that these mutants are able to maintain mtDNA (Table S5). This group is expected to include mutants lacking proteins involved in mitochondrial transcription, translation and other processes required for mitochondrial protein synthesis. Consequently, we found many deletion mutants lacking mitochondrial ribosomal subunits or tRNA synthetases (**Table 3**). Arginine prototrophy could not be restored by mating of 68 deletion mutants, suggesting loss of the [*ARG8*^*m*^] mtDNA. For 21 deletion mutants the results were intermediate, suggesting that mtDNA stability is reduced in these mutants. Combining the two latter sets of mutants, we identified 89 mutants that displayed an instable mtDNA phenotype (Table S5; see methods for details).

**TABLE 3.** **Genes required for expression of Arg8^m^ in mitochondria.** The yeast deletion collection was treated as outlined in Fig. 3A. This table is an excerpt of Table S5. Genes with known functions in arginine biosynthesis or mating and dubious ORFs were excluded from this list. Genes required for maintenance of [*ARG8*^*m*^] mtDNA are listed in Table 4.

Standard name	ORF	Standard name	ORF	Standard name	ORF
**Mitochondrial translation and RNA maturation**					
*AIM10*	*YER087W*	*MRPL13*	*YKR006C*	*MSF1*	*YPR047W*
*GEP3*	*YOR205C*	*MRPL22*	*YNL177C*	*MTG1*	*YMR097C*
*HER2*	*YMR293C*	*MRPL32*	*YCR003W*	*PET111*	*YMR257C*
*IFM1*	*YOL023W*	*MRPL38*	*YKL170W*	*PET112*	*YBL080C*
*IMG2*	*YCR071C*	*MRPL6*	*YHR147C*	*QRI5*	*YLR204W*
*MHR1*	*YDR296W*	*MRPL9*	*YGR220C*	*RSM7*	*YJR113C*
*MRF1*	*YGL143C*	*MRPS5*	*YBR251W*		
*MRM1*	*YOR201C*	*MSE1*	*YOL033W*		
**Respiratory chain components and assembly factors**					
*ATP15*	*YPL271W*	*ATP7*	*YKL016C*		
*ATP17*	*YDR377W*	*SDH4*	*YDR178W*		
**Other mitochondrial function**					
*ACO2*	*YJL200C*	*MIS1*	*YBR084W*	*POS5*	*YPL188W*
*GGC1*	*YDL198C*	*MTM1*	*YGR257C*	*SUV3*	*YPL029W*
*GRX5*	*YPL059W*	*OXA1*	*YER154W*	*TOM6*	*YOR045W*
*MDL2*	*YPL270W*	*PIM1*	*YBL022C*		
**Vacuole-related function**					
*BRO1*	*YPL084W*	*VMA16*	*YHR026W*	*VPS24*	*YKL041W*
*DID4*	*YKL002W*	*VMA21*	*YGR105W*	*VPS61*	*YDR136C*
*DOA4*	*YDR069C*	*VMA3*	*YEL027W*	*VPS63*	*YLR261C*
*SNF8*	*YPL002C*	*VMA5*	*YKL080W*		
*VAM3*	*YOR106W*	*VMA9*	*YCL005W-A*		
**Other function**					
*BIT2*	*YBR270C*	*KCS1*	*YDR017C*	*RHO4*	*YKR055W*
*COY1*	*YKL179C*	*KEX1*	*YGL203C*	*RPE1*	*YJL121C*
*CTL1*	*YMR180C*	*LCB4*	*YOR171C*	*RTS1*	*YOR014W*
*CTR9*	*YOL145C*	*LEM3*	*YNL323W*	*RTT103*	*YDR289C*
*CUS2*	*YNL286W*	*MAF1*	*YDR005C*	*SFM1*	*YOR021C*
*DAK2*	*YFL053W*	*NGL2*	*YMR285C*	*SFP1*	*YLR403W*
*EGT2*	*YNL327W*	*NPR2*	*YEL062W*	*SLY41*	*YOR307C*
*GAL10*	*YBR019C*	*OPI3*	*YJR073C*	*SNF1*	*YDR477W*
*INO2*	*YDR123C*	*PEX5*	*YDR244W*	*TFB5*	*YDR079C-A*
*INO4*	*YOL108C*	*PEX8*	*YGR077C*	*YPS7*	*YDR349C*
*IRA2*	*YOL081W*	*PIB2*	*YGL023C*		
**Unknown function**					
*FYV6*	*YNL133C*	*RRG9*	*YNL213C*	*YPL205C*	*YPL205C*
*RRG1*	*YDR065W*	*YDR114C*	*YDR114C*		

To better define the set of genes required for maintenance of mtDNA we re-analyzed these 89 mutants. We repeated the genetic analysis and again tested their growth on media lacking arginine after cytoduction with the [*ARG8*^*m*^ *rho*^*+*^] mitochondrial genome. 16 strains that did not show arginine auxotrophy were excluded from further analysis. The remaining mutants were again subjected to mating with the Δ*arg8* [*rho*^*0*^] strain, and cells were then stained with DAPI. 16 mutants showed nucleoids in fluorescence microscopy indicating that mtDNA can be maintained; i.e. they are either [*rho*^*+*^] or [*rho*^*-*^]. 57 mutants were arginine-auxotroph and devoid of mtDNA nucleoids; i.e. [*rho*^*0*^] (Table S6). We conclude that these strains are severely defective in maintenance of mtDNA (**Table 4**). This group is expected to include mutants lacking proteins involved in replication and inheritance of mtDNA. Indeed, we found mutants lacking proteins involved in mtDNA metabolism, such as Δ*mgm101*, Δ*mip1*, and Δ*rim1*. It is known that mitochondrial fusion is essential for maintenance of mtDNA [70]. In agreement with this, fusion-defective mutants Δ*fzo1*, Δ*mgm1*, Δ*pcp1*, and Δ*ugo1* were also found to be [*rho*^*0*^].

**TABLE 4.** **Genes required for maintenance of [*ARG8*^*m*^] mtDNA.** The yeast deletion collection was treated as outlined in Fig. 3A, and mutants showing an instable mtDNA phenotype were confirmed by DAPI staining. This table is an excerpt of Table S6. Genes with known functions in arginine biosynthesis or mating and dubious ORFs were excluded from this list.

Standard name	ORF	Standard name	ORF	Standard name	ORF
**mtDNA metabolism**					
*MGM101*	*YJR144W*	*MIP1*	*YOR330C*	*RIM1*	*YCR028C-A*
**Mitochondrial transcription**					
*MTF1*	*YMR228W*	*RPO41*	*YFL036W*		
**Mitochondrial translation and RNA maturation**					
*AEP3*	*YPL005W*	*MRPL17*	*YNL252C*	*MSW1*	*YDR268W*
*ATP22*	*YDR350C*	*MRPL20*	*YKR085C*	*MSY1*	*YPL097W*
*DIA4*	*YHR011W*	*MRPL23*	*YOR150W*	*PET123*	*YOR158W*
*IMG1*	*YCR046C*	*MRPL27*	*YBR282W*	*PET130*	*YJL023C*
*MRP17*	*YKL003C*	*MRPL35*	*YDR322W*	*RML2*	*YEL050C*
*MRP20*	*YDR405W*	*MRPL37*	*YBR268W*	*RRG8*	*YPR116W*
*MRP21*	*YBL090W*	*MRPL49*	*YJL096W*	*RSM18*	*YER050C*
*MRP4*	*YHL004W*	*MRPL51*	*YPR100W*	*RSM19*	*YNR037C*
*MRP51*	*YPL118W*	*MRPS12*	*YNR036C*	*RSM23*	*YGL129C*
*MRP7*	*YNL005C*	*MRPS16*	*YPL013C*	*RSM24*	*YDR175C*
*MRPL10*	*YNL284C*	*MRPS35*	*YGR165W*	*RSM27*	*YGR215W*
*MRPL11*	*YDL202W*	*MSK1*	*YNL073W*	*SWS2*	*YNL081C*
*MRPL16*	*YBL038W*	*MST1*	*YKL194C*		
**Respiratory chain components and assembly factors**					
*COX5A*	*YNL052W*				
**Mitochondrial fusion**					
*FZO1*	*YBR179C*	*PCP1*	*YGR101W*		
*MGM1*	*YOR211C*	*UGO1*	*YDR470C*		
**Other mitochondrial function**					
*MDJ1*	*YFL016C*	*OCT1*	*YKL134C*	*PPA2*	*YMR267W*
**Vacuole-related function**					
*VPS33*	*YLR396C*				
**Other function**					
*HPR1*	*YDR138W*	*RPP2A*	*YOL039W*	*UAF30*	*YOR295W*
**Unknown function**					
*LCL1*	*YPL056C*	*SOV1*	*YMR066W*		

Four previous studies [18, 71–73] have reported alternative approaches that systematically identified genes required for maintenance of mtDNA. First, a screen of 4,985 mutants of the *MAT*α deletion collection revealed 118 mutants that remained *petite* after mating with Δ*mip1* and cytoduction with wild type mtDNA [18]. Second, DAPI staining of 466 *MAT*a yeast deletions with a previously reported *pet* phenotype [24] revealed 102 mutants lacking detectable mtDNA [71]. Third, colony hybridization of 5,148 yeast deletion strains with probes specific for mtDNA and nuclear DNA revealed 180 mutants lacking mtDNA [72]. And fourth, genome sequencing of nearly all of the strains of the homozygous diploid yeast deletion collection identified 165 mutants lacking mtDNA [73]. A comparison of these screens with our results is shown in Table S7. We consider it likely that different experimental conditions and the use of different versions of the yeast deletion library account for most of the variations of the results. Furthermore, mutants that were found only once (or maybe twice) might lose their mtDNA only after prolonged growth on fermentable carbon sources and therefore are not essential for maintenance of mtDNA. These strains were largely excluded by our approach since we freshly introduced mtDNA by cytoduction and then selected for its maintenance by an auxotrophic marker.

Recent research has revealed many molecular details of mtDNA replication in yeast. It is thought to depend on the coupling of recombination, rolling circle replication, and template switching [74]. Strikingly, already 35 years ago it was found that mitochondrial protein synthesis is required for maintenance of mtDNA [75]. A dependency of mtDNA maintenance on mitochondrial translation has also been reported for the fission yeast *Schizosaccharomyces pombe* [76]. Consistently, we found that 38 deletion mutants lacking genes required for mitochondrial protein synthesis failed to maintain a functional [*ARG8*^*m*^] genome – this is by far the largest group of genes required for this process. This is in good agreement with previous studies, which also reported that mitochondrial protein synthesis is particularly important for mtDNA maintenance [18, 71]. Our results suggest that mitochondrial translation is required for mitochondrial genome maintenance even when respiratory activity is not required.

What might be the functional link between mitochondrial protein synthesis and mtDNA maintenance? The ATP synthase consists of nuclear and mitochondria-encoded subunits. The mitochondrial genes *ATP6, ATP8*, and *ATP9* encode subunits that form the proton-conducting F_o_ part. It has been shown that incomplete assembly of the ATP synthase can lead to uncoupling of the mitochondrial membrane potential, Δψ, by passive proton transport through the F_o_ part. Complete breakdown of Δψ is lethal to the cell because it impedes the import of vital proteins into the matrix. Lethality of ATPase assembly mutants can be suppressed by loss of mtDNA concomitant with loss of the proton-conducting channel [69]. It has been suggested that alterations of mitochondrial translation may frequently lead to aberrant expression of ATP synthase subunits, formation of an incorrectly assembled proton channel, and breakdown of Δψ. In this scenario, mutants retain some residual mitochondrial translation activity, and loss of mtDNA prevents synthesis of the proton channel-forming F_o_ ATP synthase subunits and thereby promotes cell survival. Thus, loss of mtDNA in mutants defective in mitochondrial translation might be a rescuing event [77].

Alternatively, mitochondrial translation might play a more active role in maintenance of mtDNA. Recently, it was discovered that mitochondrial ribosomes interact with many proteins involved in the expression of mtDNA. Together they form large assemblies that were termed ‘mitochondrial organization of gene expression‘ (MIOREX) complexes. Intriguingly, several proteins involved in mtDNA metabolism could be co-precipitated with native mitochondrial ribosomes, and a subset of MIOREX complexes was found to be associated with mtDNA nucleoids by super resolution microscopy. These observations point to an intimate connection of mtDNA maintenance and mitochondrial protein synthesis [78]. It is clear that the presence of an intact mitochondrial protein synthesis machinery is important for maintenance of the mitochondrial genome. However, the elucidation of the exact molecular mechanisms that functionally connect mitochondrial translation with maintenance of mtDNA still remains a challenge for the future.

## MATERIALS AND METHODS

### Strains, growth, and manipulation of yeast cells

Yeast strains used in this study are listed in **Table 5**. Standard methods and media were used for growth and manipulation of yeast cells [55, 79]. Replica plating of high density arrays (HDAs in 96, 384, or 1,536 colonies format) was performed using a ROTOR HDA robot (Singer Instruments, Somerset, UK). Tetrad dissection was performed with a Singer MSM Series 300 micromanipulator equipped with an Acer n30 pocket PC (Singer Instruments).

**TABLE 5.** Yeast strains used in this study.

Strain	Genotype	References
BY4741	*MAT*a *his3*Δ*1 leu2*Δ*0 met15*Δ*0 ura3*Δ*0* [*rho*^*+*^]	[43]
BY4741[rho^0^]	*MAT*a *his3*Δ*1 leu2*Δ*0 met15*Δ*0 ura3*Δ*0* [*rho*^*0*^]	This study
BY4742	*MAT*α *his3*Δ*1 leu2*Δ*0 lys2*Δ*0 ura3*Δ*0* [*rho*^*+*^]	[43]
Δ*mip1*	*MAT*α *his3*Δ*1 leu2*Δ*0 lys2*Δ*0 ura3*Δ*0* [*rho*^*0*^]	[27, 29]
J1361	*MAT*a *CEN1-16:pGal-K.lactis-URA3 kar1*Δ*15 lys2*Δ *rad5-535 leu2-3,112 can1-100 his3-11,15 trp1-1* [*rho*^*+*^]	[60]
J1361 [*rho*^*0*^]	*MAT*a *CEN1-16:pGal-K.lactis-URA3 kar1*Δ*15 lys2*Δ *rad5-535 leu2-3,112 can1-100 his3-11,15 trp1-1* [*rho*^*0*^]	This study
J1361 [A*RG8*^*m*^ *rho*^*+*^]	*MAT*a *CEN1-16:pGal-K.lactis-URA3 kar1*Δ*15 lys2*Δ *rad5-535 leu2-3,112 can1-100 his3-11,15 trp1-1* [A*RG8*^*m*^ *rho*^*+*^]	This study
J1362	*MAT*α *CEN1-16:pGal-K.lactis-URA3 kar1*Δ*15 lys2*Δ *rad5-535 leu2-3,112 can1-100 his3-11,15 trp1-1* [*rho*^*+*^]	[60]
JC8 [A*RG8*^*m*^ *rho*^*+*^]	*MAT*a *kar1-1 leu1* [*ARG8*^*m*^ *rho*^*+*^]	[50, 69, 87]
Y8205	*MAT*α *can1*Δ*::STE2pr-Sp_his5 lyp1*Δ*::STE3pr-LEU2 his3*Δ*1 leu2*Δ*0 ura3*Δ*0* [*rho*^*+*^]	[68]
YDTL88	*MAT*a *his3*Δ*1 leu2*Δ*0 met15*Δ*0 ura3*Δ*0 arg8Δ::HIS3MX6* [*rho*^*0*^]	This study
YKO strains^1^	*MAT*a *his3*Δ*1 leu2*Δ*0 met15*Δ*0 ura3*Δ*0 orf*Δ*::kanMX4* [*rho*^*+*^]	[27, 29] and this study
YKO strains [*rho*^*0*^]	*MAT*a *his3*Δ*1 leu2*Δ*0 met15*Δ*0 ura3*Δ*0 orf*Δ*::kanMX4* [*rho*^*0*^]	This study
YKO strains Δ*arg8*	*MAT*α *lyp1*Δ*::STE3pr-LEU2 his3*Δ*1 leu2*Δ*0 met15*Δ*0 ura3*Δ*0 orf*Δ*::kanMX4 arg8*Δ*::natNT2* [*rho*^*+*^]	This study
YKO strains Δ*arg8* [*rho*^*0*^]	*MAT*α *lyp1*Δ*::STE3pr-LEU2 his3*Δ*1 leu2*Δ*0 met15*Δ*0 ura3*Δ*0 orf*Δ*::kanMX4 arg8*Δ*::natNT2* [*rho*^*0*^]	This study
YKO strains Δ*arg8* [*ARG8*^*m*^ *rho*^*+*^]	*MAT*α *lyp1*Δ*::STE3pr-LEU2 his3*Δ*1 leu2*Δ*0 met15*Δ*0 ura3*Δ*0 orf*Δ*::kanMX4 arg8*Δ*::natNT2* [*ARG8*^*m*^ *rho*^*+*^]	This study
heterozygous YKO strains Δ*arg8* [*ARG8*^*m*^ *rho*^*+*^]	*MATa/*α *LYP1/lyp1*Δ*::STE3pr-LEU2 his3*Δ*1/his3*Δ*1 leu2*Δ*0/leu2*Δ*0 met15*Δ*0/met15*Δ*0 ura3*Δ*0/ura3*Δ*0 ORF/orf*Δ*::kanMX4 arg8*Δ*::natNT2/arg8*Δ*::HIS3MX6* [*ARG8*^*m*^ *rho*^*+*^]	This study
YMS001	*MAT*α *CAN1 lyp1*Δ*::STE3pr-LEU2 his3*Δ*1 leu2*Δ*0 ura3*Δ*0 met15*Δ*0* [*rho*^*+*^]	This study
YMS002	*MAT*α *CAN1 lyp1*Δ*::STE3pr-LEU2 his3*Δ*1 leu2*Δ*0 ura3*Δ*0 met15*Δ*0 arg8*Δ*::natNT2* [*rho*^*+*^]	This study
YMS003	*MAT*α *CAN1 lyp1*Δ*::STE3pr-LEU2 his3*Δ*1 leu2*Δ*0 URA3 met15*Δ*0 arg8*Δ*::natNT2* [*rho*^*+*^]	This study
YMS004	*MAT*α *his3*Δ*1 leu2*Δ*0 lys2*Δ*0 ura3*Δ*0* [pRS415 *LEU2*] [*rho*^*0*^]	This study
YMS005	*MAT*α *his3*Δ*1 leu2*Δ*0 lys2*Δ*0 ura3*Δ*0* [pRS415 *LEU2*] [*ARG8*^*m*^ *rho*^*+*^]	This study

1 Yeast knock-out strains from the yeast deletion collection

To induce loss of mtDNA, yeast strains were passaged three times on YPD plates supplemented with 50 µg/ml ethidium bromide (EtBr). In case of yeast deletion collections, this was done in HDAs in a 1,536 colonies format. Subsequent growth on YPD or YPG plates was assayed in a 384 colonies format. Absence of mtDNA was tested in randomly chosen strains by DAPI staining and fluorescence microscopy as described [80].

To delete the *ARG8* gene in a [*rho*^*0*^] strain we amplified the *HIS3MX6* cassette from plasmid pFA6a-His3MX6 [81] using primers containing sequences homologous to regions flanking the *ARG8* ORF, 5' ACA TTT TTT TCG TTT GTT AGA ATA ATT CAA GAA TCG CTA CCA ATC CGG ATC CCC GGG TTA ATT AA and 5' GAA AAA AAA AAA AAC AAT CTA TAC ATG ACA ATT TAC AAA GTA TAT GAA TTC GAG CTC GTT TAA AC, and transformed the PCR product into strain BY4741 [*rho*^*0*^] resulting in strain YDTL88.

### Cytoduction

For cytoduction with donor strains J1631, J1632, and derivatives thereof, the mtDNA donor strain was grown to a lawn on a YPD plate, transferred as a 1,536 colonies HDA to a YPD plate, and incubated for 2 days at 30°C. In parallel, the recipient strain collection was first grown on YPD plates in 384 colonies HDAs, up-arrayed to 1,536 colonies HDAs, and incubated for 2 days at 30°C. Then, donor and recipient strains were combined, incubated for 1 day at 30°C, passaged two times for 1-2 days on YPGal plates, passaged two times on SCGal plates supplemented with 50 mg/l uracil and 1 g/l 5'FOA [82] and lacking lysine, and transferred to YPD, YPG, and/or SD plates lacking arginine.

### Construction of a Δ*arg8* yeast deletion collection

The starting strain for SGA, Y8205, contains a Δ*can1* deletion to allow the use of canavanine as a selection marker [68]. Canavanine, a structural analog of arginine, is taken up into the cell by an arginine permease encoded by the *CAN1* gene and incorporated into proteins, leading to non-functional translation products [83]. As construction of a yeast deletion collection with a nuclear Δ*arg8* allele required the presence of functional Can1, we introduced a wild type *CAN1* gene to facilitate arginine uptake and a *URA3* gene as an alternative selectable marker into strain Y8205. The *URA3* containing plasmid pRS416 [84] was first transformed into BY4741 [43]. This strain was mated with Y8205, diploids were selected on SD medium lacking methionine and uracil, and cells were subjected to sporulation and tetrad dissection. We selected haploid cells that were *MAT*α, resistant to thialysine, sensitive to canavanine, and auxotroph for uracil and methionine, resulting in strain YMS001. To delete the *ARG8* gene in YMS001, we amplified the *natNT2* cassette conferring resistance to nourseothricin (NTC) from plasmid pYM-N7 [85] by PCR using primers containing sequences homologous to regions flanking the *ARG8* ORF, 5' GTG ACT GCG AAC ATT TTT TTC GTT TGT TAG AAT AAT TCA AGA ATC GCT ACC AAT CGT ACG CTG CAG GTC GAC G and 5' ATA TAA AGA TGA AAA AAA AAA AAA CAA TCT ATA CAT GAC AAT TTA CAA AGT ATA TGA GCT CGA TTA CAA CAG GTG TTG TCC. Transformation of the PCR product into YMS001 and selection of NTC-resistant clones resulted in strain YMS002. The wild type *URA3* gene was amplified from strain D273-10B [86] using primers 5' TTG ATA AGA AGA GTA TTG AGA AGG GCA ACG and 5' TAT ATA TAC GCC AGT ACA CCT TAT CGG CCC. Transformation of the PCR product into YMS002 and selection of uracil prototroph clones resulted in strain YMS003, which served as a starter strain for SGA.

The SGA to introduce the Δ*arg8* allele into the yeast deletion collection was performed essentially as described [68] with some modifications as outlined below. Strain YMS003 was plated as a lawn on YPD plates and combined with HDAs (1,536 colonies format) of the *MAT*a deletion collection on YPD plates. Diploids were selected by two passages on SD medium supplemented with G418 and lacking uracil. Sporulation was induced for 10 days at 22°C. Selection of haploids was by subsequent selection rounds on SCD supplemented with 5'FOA and thialysine and lacking leucine, SCD supplemented with 5'FOA, thialysine, and G418 and lacking leucine, and three passages on SCD supplemented with 5'FOA, thialysine, G418, and NTC and lacking leucine. To verify the replacement of *ARG8* by the Δa*rg8* allele, growth was tested on SD medium lacking arginine or uracil. 611 strains were still able to grow in the absence of arginine after the first SGA, and 590 strains after a second SGA. We combined the strains obtained in both SGAs resulting in a Δ*arg8* yeast deletion collection that allowed us to screen 4,523 deletion mutants. This corresponds to 90.9% of the 4,973 strains that were originally present in the collection. It should be noted that the Δ*arg8* deletion collection was *MAT*α because haploid selection was with the *lyp1*Δ*::STE3pr-LEU2* allele.

### Construction of a [*ARG8*^*m*^ *rho*^*+*^] cytoduction donor strain

To introduce the [*ARG8*^*m*^ *rho*^*+*^] mitochondrial genome [69] into the Δ*arg8* yeast deletion collection, a mtDNA donor was constructed as follows. Strain BY4742 was transformed with the *LEU2* marker plasmid, pRS415 [84], and cured from mtDNA by growth on EtBr-containing medium, producing strain YMS004. Strain YMS004 was mated with JC8 [A*RG8*^*m*^ *rho*^*+*^] and diploids were selected on SD minimal medium supplemented with histidine and lysine (*KAR1* x *kar1-1* crosses produce diploids with mixed parental genotypes at low frequency [87]). After sporulation and tetrad dissection strain YMS005 was obtained. Strain J1361 [60] was cured from mtDNA by growth on EtBr-containing medium and served as a recipient for mtDNA from strain YMS005. Cytoductants were selected on YPG medium supplemented with canavanine. The resulting strain, J1361 [*ARG8*^*m*^ *rho*^*+*^], was used as a mtDNA donor strain for cytoduction with the Δ*arg8* [*rho*^*0*^] yeast deletion collection.

### Screen for maintenance of [*ARG8*^*m*^] mtDNA

The screen for maintenance of [*ARG8*^*m*^] mtDNA was performed exclusively on media containing fermentable carbon sources. High density arrays in 384 or 1,536 colonies format were produced using a ROTOR HDA robot according to the scheme outlined in **Fig. 3A**. Growth of the individual strains was manually assessed. Biological replicates resulting from two Δ*arg8* SGAs (see above) were analyzed on the same plates with two technical replicates for each deletion mutant. Strains that were lost at any step of the replica plating or that retained arginine prototrophy after the Δ*arg8* SGAs were omitted from further analysis. Only strains that were not rescued by cytoduction with the [*ARG8*^*m*^] mtDNA were scored for growth after mating with the Δ*arg8* [*rho*^*0*^] strain. These strains were classified into three categories: “rescue” indicates that growth on plates lacking arginine was restored, “no rescue” means that the strains remained arginine auxotroph after mating with the Δ*arg8* [*rho*^*0*^] strain, and “ambiguous” refers to strains where the resulting phenotype was intermediate (see column “Screen result” in Table S5). The latter class also includes strains that showed opposite behavior between biological replicates, i.e. growth and no growth after mating with Δ*arg8* [*rho*^*0*^]. For each strain it is indicated in Table S5 whether both biological replicates were analyzed and whether they behaved identically. If at least one biological replicate showed arginine auxotrophy after mating with Δ*arg8* [*rho*^*0*^] the deletion mutant was considered to have an instable mtDNA phenotype.

### Venn diagrams

Venn diagrams were generated using the online tool ‘Venny' as described by Oliveros, J.C. (2007-2015) Venny. An interactive tool for comparing lists with Venn's diagrams. https://bioinfogp.cnb.csic.es/tools/venny/index.html

## SUPPLEMENTAL MATERIAL

Click here for supplemental data file.

Click here for supplemental data file.

All supplemental data for this article are available online at http://www.microbialcell.com/researcharticles/2020a-stenger-microbial-cell/.

## Abbreviations:

5'FOA: – 5-fluoroorotic acid;
EtBr: – ethidium bromide;
MIOREX: – mitochondrial organization of gene expression;
mtDNA: – mitochondrial DNA;
SGA: – synthetic genetic array;
vATPase: – vacuolar ATPase.
