# Supplementary material for: Systematic analysis of nuclear gene function in respiratory growth and expression of the mitochondrial genome in S. cerevisiae
Source: Microb Cell. 2020 Jun 30;7(9):234–49. doi: 10.15698/mic2020.09.729 (PMC7453639; doi:10.15698/mic2020.09.729)
Supplement: Supplementary file 1 [file mic-07-234-s01.pdf]

SUPPLEMENTAL TABLES

Systematic analysis of nuclear gene function in respiratory growth and expression of the mitochondrial genome in *S. cerevisiae*

Maria Stenger, Duc Tung Le, Till Klecker, Benedikt Westermann

Zellbiologie, Universität Bayreuth, 95440 Bayreuth, Germany

Table S1. Combined results of four genome-wide screens to obtain high confidence *pet* genes. This table contains all ORFs and dubious ORFs from all non-mitochondrial chromosomes. The list has been taken from the Saccharomyces Genome Database, SGD, (www.yeastgenome.org; [36]) on September 25, 2019. For each ORF, the systematic gene name, the standard gene name and a brief description of the protein function according to SGD are given. Furthermore, this list contains the presence of the protein in a high confidence mitochondrial proteome [8], the results of the four *pet* screens ([18, 23, 25] and this work), and the *pet* score. The value "nd" indicates that the deletion mutant has not been tested (in most cases because it was missing from the deletion collection that was used for the screen). Some ORFs have been renamed or merged. In this case, the ORF name from the original dataset has been updated to the new nomenclature.

<u>Table S1 is supplied as a separate Excel file.</u>

References

8. Morgenstern M, Stiller SB, Lubbert P, Peikert CD, Dannenmaier S, Drepper F, Weill U, Hoss P, Feuerstein R, Gebert M, Bohnert M, van der Laan M, Schuldiner M, Schutze C, Oeljeklaus S, Pfanner N, Wiedemann N, Warscheid B (**2017**). Definition of a high-confidence mitochondrial proteome at quantitative scale. **Cell Rep** 19(13): 2836-2852. doi: 10.1016/j.celrep.2017.06.014.

18. Merz S, Westermann B (**2009**). Genome-wide deletion mutant analysis reveals genes required for respiratory growth, mitochondrial genome maintenance and mitochondrial protein synthesis in *Saccharomyces cerevisiae*. **Genome Biol** 10(9): R95. doi: 10.1186/gb-2009-10-9-r95.

23. Dimmer KS, Fritz S, Fuchs F, Messerschmitt M, Weinbach N, Neupert W, Westermann B (**2002**). Genetic basis of mitochondrial function and morphology in *Saccharomyces cerevisiae*. **Mol Biol Cell** 13(3): 847-853. doi: 10.1091/mbc.01-12-0588.

25. Luban C, Beutel M, Stahl U, Schmidt U (**2005**). Systematic screening of nuclear encoded proteins involved in the splicing metabolism of group II introns in yeast mitochondria. **Gene** 354(72-79. doi: 10.1016/j.gene.2005.03.023.

36. Cherry JM, Hong EL, Amundsen C, Balakrishnan R, Binkley G, Chan ET, Christie KR, Costanzo MC, Dwight SS, Engel SR, Fisk DG, Hirschman JE, Hitz BC, Karra K, Krieger CJ, Miyasato SR, Nash RS, Park J, Skrzypek MS, Simison M, Weng S, Wong ED (**2012**). Saccharomyces Genome Database: the genomics resource of budding yeast. **Nucleic Acids Res** 40(Database issue): D700-705. doi: 10.1093/nar/gkr1029.

Table S2. High confidence *pet* genes in yeast. This table contains the systematic gene name according to SGD, the standard gene name according to SGD, the presence of the encoded protein in a high confidence mitochondrial proteome [8], the *pet* score, and for dubious ORFs a list of overlapping genomic features according to SGD, including their respective *pet* score (overlapping dubious ORFs have not been annotated). This table is an excerpt of data compiled in Table S1. Genes were manually grouped into functional categories.

Reference

8. Morgenstern M, Stiller SB, Lubbert P, Peikert CD, Dannenmaier S, Drepper F, Weill U, Hoss P, Feuerstein R, Gebert M, Bohnert M, van der Laan M, Schuldiner M, Schutze C, Oeljeklaus S, Pfanner N, Wiedemann N, Warscheid B (**2017**). Definition of a high-confidence mitochondrial proteome at quantitative scale. **Cell Rep** 19(13): 2836-2852. doi: 10.1016/j.celrep.2017.06.014.

Stenger et al. - Table S2

ORF	standard name	Morgenstern et al. (2017)	*pet* score	overlapping ORFs
**Mitochondrial translation and RNA maturation**				
YBL038W	*MRPL16*	mitochondrial	0.75	
YBL080C	*PET112*	mitochondrial	0.75	
YBL090W	*MRP21*	mitochondrial	1.00	
YBR122C	*MRPL36*	mitochondrial	1.00	
YBR251W	*MRPS5*	mitochondrial	0.75	
YBR268W	*MRPL37*	mitochondrial	0.75	
YBR282W	*MRPL27*	mitochondrial	1.00	
YCR003W	*MRPL32*	mitochondrial	1.00	
YCR024C	*SLM5*	mitochondrial	1.00	
YCR046C	*IMG1*	mitochondrial	1.00	
YCR071C	*IMG2*	mitochondrial	1.00	
YDL045W-A	*MRP10*	mitochondrial	0.75	
YDL069C	*CBS1*	mitochondrial	0.75	
YDL202W	*MRPL11*	mitochondrial	0.75	
YDR115W	*MRX14*	mitochondrial	0.75	
YDR175C	*RSM24*	mitochondrial	1.00	
YDR197W	*CBS2*	mitochondrial	0.75	
YDR237W	*MRPL7*	mitochondrial	0.75	
YDR268W	*MSW1*	mitochondrial	0.75	
YDR296W	*MHR1*	mitochondrial	0.75	
YDR337W	*MRPS28*	mitochondrial	0.75	
YDR347W	*MRP1*	mitochondrial	0.75	
YDR350C	*ATP22*	mitochondrial	0.75	
YDR405W	*MRP20*	mitochondrial	0.75	
YEL050C	*RML2*	mitochondrial	0.75	
YER050C	*RSM18*	mitochondrial	1.00	
YER087W	*AIM10*	mitochondrial	1.00	
YER153C	*PET122*	mitochondrial	0.75	
YGL107C	*RMD9*	mitochondrial	0.75	
YGL129C	*RSM23*	mitochondrial	1.00	
YGL143C	*MRF1*	mitochondrial	1.00	
YGR076C	*MRPL25*	mitochondrial	0.75	
YGR150C	*CCM1*	mitochondrial	1.00	
YGR171C	*MSM1*	mitochondrial	0.75	
YGR215W	*RSM27*	mitochondrial	1.00	
YGR220C	*MRPL9*	mitochondrial	1.00	
YGR222W	*PET54*	mitochondrial	1.00	
YHL004W	*MRP4*	mitochondrial	1.00	
YHL038C	*CBP2*	mitochondrial	1.00	
YHR011W	*DIA4*	mitochondrial	1.00	
YHR038W	*RRF1*	mitochondrial	0.75	
YHR091C	*MSR1*	mitochondrial	1.00	
YHR147C	*MRPL6*	mitochondrial	1.00	
YHR168W	*MTG2*	mitochondrial	0.75	
YIR021W	*MRS1*	mitochondrial	0.75	
YJL063C	*MRPL8*	mitochondrial	0.75	
YJL096W	*MRPL49*	mitochondrial	1.00	

ORF	standard name	Morgenstern et al. (2017)	*pet* score	overlapping ORFs
YJL102W	*MEF2*	mitochondrial	0.75	
YJL209W	*CBP1*	mitochondrial	0.75	
YJR113C	*RSM7*	mitochondrial	1.00	
YKL003C	*MRP17*	mitochondrial	1.00	
YKL138C	*MRPL31*	mitochondrial	0.75	
YKL155C	*RSM22*	mitochondrial	1.00	
YKL170W	*MRPL38*	mitochondrial	1.00	
YKL194C	*MST1*	mitochondrial	1.00	
YKR006C	*MRPL13*	mitochondrial	1.00	
YKR085C	*MRPL20*	mitochondrial	0.75	
YLR067C	*PET309*	mitochondrial	1.00	
YLR069C	*MEF1*	mitochondrial	0.75	
YLR091W	*GEP5*	mitochondrial	0.75	
YLR139C	*SLS1*	mitochondrial	1.00	
YLR203C	*MSS51*	mitochondrial	1.00	
YLR312W-A	*MRPL15*	mitochondrial	1.00	
YLR382C	*NAM2*	mitochondrial	0.75	
YLR439W	*MRPL4*	mitochondrial	0.75	
YMR064W	*AEP1*	mitochondrial	1.00	
YMR097C	*MTG1*	mitochondrial	0.75	
YMR098C	*ATP25*	mitochondrial	1.00	
YMR158W	*MRPS8*	mitochondrial	0.75	
YMR193W	*MRPL24*	mitochondrial	0.75	
YMR257C	*PET111*	mitochondrial	1.00	
YMR282C	*AEP2*	mitochondrial	0.75	
YMR286W	*MRPL33*	mitochondrial	1.00	
YMR293C	*HER2*	mitochondrial	1.00	
YNL005C	*MRP7*	mitochondrial	1.00	
YNL073W	*MSK1*	mitochondrial	1.00	
YNL081C	*SWS2*	mitochondrial	1.00	
YNL177C	*MRPL22*	mitochondrial	1.00	
YNL185C	*MRPL19*	mitochondrial	1.00	
YNL252C	*MRPL17*	mitochondrial	0.75	
YNL284C	*MRPL10*	mitochondrial	0.75	
YNR036C	*MRPS12*	mitochondrial	0.75	
YNR037C	*RSM19*	mitochondrial	1.00	
YNR045W	*PET494*	mitochondrial	0.75	
YOL023W	*IFM1*	mitochondrial	0.75	
YOL033W	*MSE1*	mitochondrial	1.00	
YOR150W	*MRPL23*	mitochondrial	1.00	
YOR158W	*PET123*	mitochondrial	1.00	
YOR187W	*TUF1*	mitochondrial	1.00	
YOR205C	*GEP3*	mitochondrial	0.75	
YOR350C	*MNE1*	mitochondrial	0.75	
YPL005W	*AEP3*	mitochondrial	0.75	
YPL013C	*MRPS16*	mitochondrial	1.00	
YPL097W	*MSY1*	mitochondrial	1.00	
YPL104W	*MSD1*	mitochondrial	0.75	
YPL118W	*MRP51*	mitochondrial	0.75	

ORF	standard name	Morgenstern et al. (2017)	*pet* score	overlapping ORFs
YPL173W	*MRPL40*	mitochondrial	1.00	
YPR047W	*MSF1*	mitochondrial	0.75	
YPR100W	*MRPL51*	mitochondrial	0.75	
YPR116W	*RRG8*	mitochondrial	1.00	
**Mitochondrial transcription**				
YDL044C	*MTF2*	mitochondrial	0.75	
YDR194C	*MSS116*	mitochondrial	0.75	
YFL036W	*RPO41*	mitochondrial	0.75	
YMR228W	*MTF1*	mitochondrial	1.00	
**Respiratory chain components and assembly factors**				
YAL039C	*CYC3*	mitochondrial	0.75	
YBL045C	*COR1*	mitochondrial	1.00	
YBL099W	*ATP1*	mitochondrial	1.00	
YBR037C	*SCO1*	mitochondrial	0.75	
YBR039W	*ATP3*	mitochondrial	1.00	
YDL067C	*COX9*	mitochondrial	1.00	
YDL107W	*MSS2*	mitochondrial	1.00	
YDR079W	*PET100*	mitochondrial	1.00	
YDR231C	*COX20*	mitochondrial	0.75	
YDR298C	*ATP5*	mitochondrial	1.00	
YDR375C	*BCS1*	mitochondrial	0.75	
YDR377W	*ATP17*	mitochondrial	1.00	
YDR529C	*QCR7*	mitochondrial	0.75	
YEL024W	*RIP1*	mitochondrial	1.00	
YER058W	*PET117*	mitochondrial	0.75	
YGR062C	*COX18*	mitochondrial	1.00	
YGR112W	*SHY1*	mitochondrial	1.00	
YGR174C	*CBP4*	mitochondrial	0.75	
YHR051W	*COX6*	mitochondrial	1.00	
YJL166W	*QCR8*	mitochondrial	0.75	
YJL180C	*ATP12*	mitochondrial	1.00	
YJR121W	*ATP2*	mitochondrial	0.75	
YKL016C	*ATP7*	mitochondrial	1.00	
YKL087C	*CYT2*	mitochondrial	0.75	
YKL148C	*SDH1*	mitochondrial	0.75	
YLL018C-A	*COX19*	mitochondrial	1.00	
YLL041C	*SDH2*	mitochondrial	0.75	
YLR038C	*COX12*	mitochondrial	1.00	
YLR393W	*ATP10*	mitochondrial	0.75	
YMR035W	*IMP2*	mitochondrial	0.75	
YMR150C	*IMP1*	mitochondrial	1.00	
YMR256C	*COX7*	mitochondrial	1.00	
YNL052W	*COX5A*	mitochondrial	1.00	
YNL315C	*ATP11*	mitochondrial	1.00	
YOL071W	*SDH5*	mitochondrial	0.75	
YOR065W	*CYT1*	mitochondrial	1.00	
YPL078C	*ATP4*	mitochondrial	1.00	
YPL132W	*COX11*	mitochondrial	0.75	
YPL172C	*COX10*	mitochondrial	1.00	

ORF	standard name	Morgenstern et al. (2017)	*pet* score	overlapping ORFs
YPL215W	*CBP3*	mitochondrial	0.75	
YPL271W	*ATP15*	mitochondrial	1.00	
YPR191W	*QCR2*	mitochondrial	0.75	
**mtDNA metabolism**				
YCR028C-A	*RIM1*	mitochondrial	1.00	
YHR120W	*MSH1*	mitochondrial	1.00	
YJR144W	*MGM101*	mitochondrial	1.00	
YML061C	*PIF1*	mitochondrial	0.75	
YMR072W	*ABF2*	mitochondrial	1.00	
YOL095C	*HMI1*	mitochondrial	0.75	
YOR330C	*MIP1*	mitochondrial	1.00	
**Other mitochondrial proteins**				
YAL044C	*GCV3*	mitochondrial	1.00	
YBR003W	*COQ1*	mitochondrial	1.00	
YBR026C	*ETR1*	mitochondrial	1.00	
YBR179C	*FZO1*	mitochondrial	0.75	
YDL198C	*GGC1*	mitochondrial	0.75	
YDR148C	*KGD2*	mitochondrial	1.00	
YDR204W	*COQ4*	mitochondrial	0.75	
YEL059C-A	*SOM1*	mitochondrial	0.67	
YER014W	*HEM14*	mitochondrial	0.67	
YER017C	*AFG3*	mitochondrial	0.75	
YER061C	*CEM1*	mitochondrial	1.00	
YER145C	*FTR1*	mitochondrial	0.75	
YER154W	*OXA1*	mitochondrial	1.00	
YFL016C	*MDJ1*	mitochondrial	1.00	
YFL018C	*LPD1*	mitochondrial	0.75	
YGR255C	*COQ6*	mitochondrial	0.75	
YHR067W	*HTD2*	mitochondrial	1.00	
YJL046W	*AIM22*	mitochondrial	1.00	
YJR122W	*IBA57*	mitochondrial	1.00	
YKL040C	*NFU1*	mitochondrial	0.75	
YKL055C	*OAR1*	mitochondrial	0.75	
YKL134C	*OCT1*	mitochondrial	1.00	
YLL027W	*ISA1*	mitochondrial	1.00	
YLR201C	*COQ9*	mitochondrial	0.75	
YLR239C	*LIP2*	mitochondrial	0.75	
YLR304C	*ACO1*	mitochondrial	0.75	
YLR369W	*SSQ1*	mitochondrial	0.75	
YML110C	*COQ5*	mitochondrial	0.75	
YMR089C	*YTA12*	mitochondrial	1.00	
YMR267W	*PPA2*	mitochondrial	1.00	
YMR287C	*DSS1*	mitochondrial	0.75	
YNR041C	*COQ2*	mitochondrial	1.00	
YOL008W	*COQ10*	mitochondrial	0.75	
YOL096C	*COQ3*	mitochondrial	0.75	
YOR125C	*CAT5*	mitochondrial	0.75	
YOR211C	*MGM1*	mitochondrial	1.00	
YOR221C	*MCT1*	mitochondrial	0.75	

ORF	standard name	Morgenstern et al. (2017)	*pet* score	overlapping ORFs
YOR241W	*MET7*	mitochondrial	1.00	
YPL029W	*SUV3*	mitochondrial	0.75	
YPL059W	*GRX5*	mitochondrial	0.75	
YPL148C	*PPT2*	mitochondrial	0.75	
YPL262W	*FUM1*	mitochondrial	0.75	
YPR067W	*ISA2*	mitochondrial	1.00	
**Vacuole-related function**				
YCL005W-A	*VMA9*		1.00	
YDL185W	*VMA1*		1.00	
YEL027W	*VMA3*		0.75	
YEL051W	*VMA8*		0.75	
YGR105W	*VMA21*		1.00	
YHR026W	*VMA16*		0.75	
YHR039C-A	*VMA10*		0.75	
YHR060W	*VMA22*		0.75	
YKL002W	*DID4*		0.75	
YKL080W	*VMA5*		1.00	
YLR396C	*VPS33*		0.75	
YLR447C	*VMA6*		1.00	
YOR332W	*VMA4*		0.75	
YPL045W	*VPS16*		0.75	
YPL234C	*VMA11*		0.75	
**Other proteins**				
YAL012W	*CYS3*		0.75	
YAL016W	*TPD3*		0.75	
YBL021C	*HAP3*		0.75	
YDR477W	*SNF1*		0.75	
YER014C-A	*BUD25*		0.75	*HEM14* (*pet* score: 0.67) and *FAA2* (*pet* score: 0.00)
YGL070C	*RPB9*		0.75	
YGL071W	*AFT1*		1.00	
YGL115W	*SNF4*		0.75	
YGL135W	*RPL1B*		0.75	
YGL237C	*HAP2*		0.75	
YGL240W	*DOC1*		0.75	
YGR180C	*RNR4*		1.00	
YJL176C	*SWI3*		1.00	
YJR090C	*GRR1*		0.75	
YKL054C	*DEF1*		0.75	
YKL109W	*HAP4*		1.00	
YLR240W	*VPS34*		0.75	
YLR260W	*LCB5*		0.75	
YLR377C	*FBP1*		0.75	
YMR021C	*MAC1*		0.75	
YOR358W	*HAP5*		1.00	
YPR124W	*CTR1*		0.75	
**Unknown function**				
YDR065W	*RRG1*	mitochondrial	1.00	
YDR114C			1.00	*MRX14* (*pet* score: 0.75)

ORF	standard name	Morgenstern et al. (2017)	*pet* score	overlapping ORFs
YLL033W	*IRC19*	mitochondrial	0.75	
YMR066W	*SOV1*	mitochondrial	0.75	
YNL184C			1.00	*MRPL19* (*pet* score: 1.00)
YNL213C	*RRG9*	mitochondrial	1.00	
YOR305W	*RRG7*	mitochondrial	1.00	
**Dubious ORFs**				
YBL100C			0.75	*ATP1* (*pet* score: 1.00)
YCL007C			1.00	*VMA9* (*pet* score: 1.00)
YDL068W			0.75	*CBS1* (*pet* score: 0.75)
YDR230W			1.00	*COX20* (*pet* score: 0.75)
YGL218W			0.75	*MDM34* (*pet* score: 0.00)
YGR219W			0.75	*MRPL9* (*pet* score: 1.00)
YJL120W			0.75	*RPE1* (*pet* score: 0.50)
YJR114W			1.00	*RSM7* (*pet* score: 1.00)
YKL169C			1.00	*MRPL38* (*pet* score: 1.00)
YLR202C			1.00	*MSS51* (*pet* score: 1.00) and *COQ9* (*pet* score: 0.75)
YNL170W			0.75	*PSD1* (*pet* score: 0.00)
YOR200W			1.00	*MRM1* (*pet* score: 0.50)
YOR331C			1.00	*VMA4* (*pet* score: 0.75)
YPR099C			0.75	*MRPL51* (*pet* score: 0.75)

Table S3. Results of the cytoduction screen. This table contains the systematic gene name according to SGD, the standard gene name according to SGD, and the *pet* score. Class I mutants showed a *pet* phenotype before and after cytoduction, class II mutants showed a *pet* phenotype only before cytoduction and were rescued by replenishment of intact mtDNA, and class III mutants acquired a *pet* phenotype only after cytoduction. It is indicated whether this behavior was reproduced in an independent experiment.

Stenger et al. - Table S3

ORF	standard name	*pet* score	reproducible	class
YAL016W	*TPD3*	0.75	no	class I
YBL022C	*PIM1*	0.50	yes	class I
YBL038W	*MRPL16*	0.75	yes	class I
YBL045C	*COR1*	1.00	yes	class I
YBL080C	*PET112*	0.75	no	class I
YBL090W	*MRP21*	1.00	yes	class I
YBR003W	*COQ1*	1.00	yes	class I
YBR037C	*SCO1*	0.75	yes	class I
YBR120C	*CBP6*	0.50	yes	class I
YBR179C	*FZO1*	0.75	yes	class I
YBR251W	*MRPS5*	0.75	yes	class I
YBR268W	*MRPL37*	0.75	yes	class I
YBR282W	*MRPL27*	1.00	yes	class I
YCL005W-A	*VMA9*	1.00	yes	class I
YCL007C		1.00	yes	class I
YCR003W	*MRPL32*	1.00	yes	class I
YCR024C	*SLM5*	1.00	yes	class I
YCR028C-A	*RIM1*	1.00	yes	class I
YCR071C	*IMG2*	1.00	yes	class I
YDL067C	*COX9*	1.00	yes	class I
YDL069C	*CBS1*	0.75	yes	class I
YDL072C	*YET3*	0.25	no	class I
YDL107W	*MSS2*	1.00	yes	class I
YDL167C	*NRP1*	0.50	yes	class I
YDL185W	*VMA1*	1.00	yes	class I
YDL198C	*GGC1*	0.75	yes	class I
YDL202W	*MRPL11*	0.75	yes	class I
YDR065W	*RRG1*	1.00	yes	class I
YDR079C-A	*TFB5*	0.33	no	class I
YDR079W	*PET100*	1.00	yes	class I
YDR114C		1.00	yes	class I
YDR138W	*HPR1*	0.25	yes	class I
YDR148C	*KGD2*	1.00	yes	class I
YDR175C	*RSM24*	1.00	yes	class I
YDR230W		1.00	yes	class I
YDR231C	*COX20*	0.75	yes	class I
YDR268W	*MSW1*	0.75	yes	class I
YDR298C	*ATP5*	1.00	yes	class I
YDR300C	*PRO1*	0.25	yes	class I
YDR322W	*MRPL35*	0.50	yes	class I
YDR347W	*MRP1*	0.75	yes	class I
YDR350C	*ATP22*	0.75	yes	class I
YDR375C	*BCS1*	0.75	yes	class I
YDR377W	*ATP17*	1.00	yes	class I
YDR405W	*MRP20*	0.75	yes	class I
YDR470C	*UGO1*	0.25	yes	class I
YDR477W	*SNF1*	0.75	no	class I
YDR529C	*QCR7*	0.75	yes	class I

ORF	standard name	*pet* score	reproducible	class
YEL024W	*RIP1*	1.00	yes	class I
YEL050C	*RML2*	0.75	yes	class I
YEL059C-A	*SOM1*	0.67	yes	class I
YER014W	*HEM14*	0.67	yes	class I
YER050C	*RSM18*	1.00	yes	class I
YER058W	*PET117*	0.75	yes	class I
YER087W	*AIM10*	1.00	yes	class I
YER153C	*PET122*	0.75	yes	class I
YER154W	*OXA1*	1.00	yes	class I
YFL016C	*MDJ1*	1.00	yes	class I
YFL018C	*LPD1*	0.75	no	class I
YFL036W	*RPO41*	0.75	yes	class I
YGL071W	*AFT1*	1.00	no	class I
YGL095C	*VPS45*	0.50	no	class I
YGL119W	*COQ8*	0.33	yes	class I
YGL129C	*RSM23*	1.00	yes	class I
YGL143C	*MRF1*	1.00	yes	class I
YGR062C	*COX18*	1.00	yes	class I
YGR101W	*PCP1*	0.50	yes	class I
YGR105W	*VMA21*	1.00	yes	class I
YGR112W	*SHY1*	1.00	yes	class I
YGR150C	*CCM1*	1.00	yes	class I
YGR165W	*MRPS35*	0.50	yes	class I
YGR180C	*RNR4*	1.00	no	class I
YGR215W	*RSM27*	1.00	yes	class I
YGR219W		0.75	yes	class I
YGR220C	*MRPL9*	1.00	yes	class I
YGR222W	*PET54*	1.00	yes	class I
YGR255C	*COQ6*	0.75	yes	class I
YGR257C	*MTM1*	0.50	yes	class I
YHL004W	*MRP4*	1.00	no	class I
YHL038C	*CBP2*	1.00	yes	class I
YHR011W	*DIA4*	1.00	yes	class I
YHR026W	*VMA16*	0.75	yes	class I
YHR051W	*COX6*	1.00	yes	class I
YHR067W	*HTD2*	1.00	no	class I
YHR091C	*MSR1*	1.00	yes	class I
YHR116W	*COX23*	0.50	yes	class I
YHR147C	*MRPL6*	1.00	yes	class I
YJL023C	*PET130*	0.50	yes	class I
YJL046W	*AIM22*	1.00	yes	class I
YJL096W	*MRPL49*	1.00	yes	class I
YJL166W	*QCR8*	0.75	yes	class I
YJL176C	*SWI3*	1.00	yes	class I
YJL184W	*GON7*	0.50	no	class I
YJL209W	*CBP1*	0.75	yes	class I
YJR090C	*GRR1*	0.75	yes	class I
YJR113C	*RSM7*	1.00	yes	class I
YJR114W		1.00	yes	class I

ORF	standard name	*pet* score	reproducible	class
YJR120W		0.50	yes	class I
YJR122W	*IBA57*	1.00	yes	class I
YJR144W	*MGM101*	1.00	yes	class I
YKL003C	*MRP17*	1.00	yes	class I
YKL016C	*ATP7*	1.00	yes	class I
YKL080W	*VMA5*	1.00	yes	class I
YKL109W	*HAP4*	1.00	no	class I
YKL134C	*OCT1*	1.00	yes	class I
YKL148C	*SDH1*	0.75	no	class I
YKL155C	*RSM22*	1.00	yes	class I
YKL169C		1.00	yes	class I
YKL170W	*MRPL38*	1.00	yes	class I
YKL194C	*MST1*	1.00	yes	class I
YKR006C	*MRPL13*	1.00	yes	class I
YLL018C-A	*COX19*	1.00	yes	class I
YLL027W	*ISA1*	1.00	yes	class I
YLR038C	*COX12*	1.00	yes	class I
YLR067C	*PET309*	1.00	yes	class I
YLR139C	*SLS1*	1.00	yes	class I
YLR201C	*COQ9*	0.75	yes	class I
YLR202C		1.00	yes	class I
YLR203C	*MSS51*	1.00	yes	class I
YLR239C	*LIP2*	0.75	yes	class I
YLR240W	*VPS34*	0.75	yes	class I
YLR312W-A	*MRPL15*	1.00	yes	class I
YLR368W	*MDM30*	0.25	yes	class I
YLR396C	*VPS33*	0.75	yes	class I
YLR447C	*VMA6*	1.00	yes	class I
YML110C	*COQ5*	0.75	yes	class I
YMR021C	*MAC1*	0.75	yes	class I
YMR035W	*IMP2*	0.75	yes	class I
YMR064W	*AEP1*	1.00	yes	class I
YMR066W	*SOV1*	0.75	yes	class I
YMR098C	*ATP25*	1.00	yes	class I
YMR150C	*IMP1*	1.00	yes	class I
YMR228W	*MTF1*	1.00	yes	class I
YMR256C	*COX7*	1.00	yes	class I
YMR257C	*PET111*	1.00	yes	class I
YMR267W	*PPA2*	1.00	yes	class I
YMR286W	*MRPL33*	1.00	yes	class I
YMR293C	*HER2*	1.00	yes	class I
YNL005C	*MRP7*	1.00	yes	class I
YNL073W	*MSK1*	1.00	yes	class I
YNL081C	*SWS2*	1.00	yes	class I
YNL177C	*MRPL22*	1.00	yes	class I
YNL184C		1.00	yes	class I
YNL213C	*RRG9*	1.00	yes	class I
YNL284C	*MRPL10*	0.75	yes	class I
YNR036C	*MRPS12*	0.75	yes	class I

ORF	standard name	*pet* score	reproducible	class
YNR037C	*RSM19*	1.00	yes	class I
YNR041C	*COQ2*	1.00	yes	class I
YNR045W	*PET494*	0.75	yes	class I
YOL033W	*MSE1*	1.00	yes	class I
YOL071W	*SDH5*	0.75	yes	class I
YOL096C	*COQ3*	0.75	yes	class I
YOR065W	*CYT1*	1.00	yes	class I
YOR125C	*CAT5*	0.75	yes	class I
YOR150W	*MRPL23*	1.00	yes	class I
YOR158W	*PET123*	1.00	yes	class I
YOR187W	*TUF1*	1.00	yes	class I
YOR199W		0.50	no	class I
YOR200W		1.00	yes	class I
YOR201C	*MRM1*	0.50	yes	class I
YOR205C	*GEP3*	0.75	yes	class I
YOR211C	*MGM1*	1.00	yes	class I
YOR330C	*MIP1*	1.00	yes	class I
YOR331C		1.00	yes	class I
YOR350C	*MNE1*	0.75	yes	class I
YOR358W	*HAP5*	1.00	yes	class I
YPL005W	*AEP3*	0.75	yes	class I
YPL013C	*MRPS16*	1.00	yes	class I
YPL029W	*SUV3*	0.75	yes	class I
YPL059W	*GRX5*	0.75	yes	class I
YPL078C	*ATP4*	1.00	yes	class I
YPL097W	*MSY1*	1.00	yes	class I
YPL118W	*MRP51*	0.75	yes	class I
YPL172C	*COX10*	1.00	yes	class I
YPL173W	*MRPL40*	1.00	yes	class I
YPL262W	*FUM1*	0.75	yes	class I
YPL271W	*ATP15*	1.00	yes	class I
YPR047W	*MSF1*	0.75	yes	class I
YPR066W	*UBA3*	0.50	no	class I
YPR067W	*ISA2*	1.00	yes	class I
YPR100W	*MRPL51*	0.75	yes	class I
YPR116W	*RRG8*	1.00	yes	class I
YPR124W	*CTR1*	0.75	yes	class I
YAL012W	*CYS3*	0.75	yes	class II
YAL044C	*GCV3*	1.00	yes	class II
YAL048C	*GEM1*	0.50	yes	class II
YBL002W	*HTB2*	0.50	yes	class II
YBL044W		0.50	yes	class II
YBR026C	*ETR1*	1.00	yes	class II
YBR122C	*MRPL36*	1.00	yes	class II
YCR004C	*YCP4*	0.50	yes	class II
YCR046C	*IMG1*	1.00	no	class II
YDL032W		0.50	yes	class II
YDL040C	*NAT1*	0.25	yes	class II
YDL063C	*SYO1*	0.50	yes	class II

ORF	standard name	*pet* score	reproducible	class
YDL133C-A	*RPL41B*	0.25	yes	class II
YDL146W	*LDB17*	0.50	yes	class II
YDL206W		0.25	yes	class II
YDR042C		0.50	yes	class II
YDR078C	*SHU2*	0.50	yes	class II
YDR116C	*MRPL1*	0.50	yes	class II
YDR178W	*SDH4*	0.25	yes	class II
YDR493W	*MZM1*	0.25	yes	class II
YDR507C	*GIN4*	0.50	yes	class II
YDR512C	*EMI1*	0.50	yes	class II
YDR518W	*EUG1*	0.50	yes	class II
YDR521W		0.25	yes	class II
YDR523C	*SPS1*	0.50	yes	class II
YEL036C	*ANP1*	0.25	yes	class II
YER061C	*CEM1*	1.00	yes	class II
YER103W	*SSA4*	0.50	yes	class II
YER110C	*KAP123*	0.50	yes	class II
YER122C	*GLO3*	0.50	yes	class II
YER169W	*RPH1*	0.50	yes	class II
YFR048W	*RMD8*	0.25	yes	class II
YGL084C	*GUP1*	0.25	yes	class II
YGL107C	*RMD9*	0.75	no	class II
YGL115W	*SNF4*	0.75	yes	class II
YGL198W	*YIP4*	0.25	yes	class II
YGL220W	*BOL2*	0.50	yes	class II
YGL240W	*DOC1*	0.75	yes	class II
YGR157W	*CHO2*	0.25	yes	class II
YGR174C	*CBP4*	0.75	yes	class II
YGR183C	*QCR9*	0.50	yes	class II
YHR050W-A		0.33	yes	class II
YHR120W	*MSH1*	1.00	yes	class II
YHR175W-A		0.33	yes	class II
YIL012W		0.25	yes	class II
YIL041W	*GVP36*	0.25	yes	class II
YJL027C		0.25	yes	class II
YJR004C	*SAG1*	0.50	yes	class II
YJR060W	*CBF1*	0.25	yes	class II
YJR150C	*DAN1*	0.25	yes	class II
YKL002W	*DID4*	0.75	no	class II
YKL040C	*NFU1*	0.75	yes	class II
YKL054C	*DEF1*	0.75	yes	class II
YKL087C	*CYT2*	0.75	yes	class II
YKL137W	*CMC1*	0.50	yes	class II
YKL208W	*CBT1*	0.50	no	class II
YKL212W	*SAC1*	0.50	yes	class II
YLL006W	*MMM1*	0.50	yes	class II
YLL041C	*SDH2*	0.75	yes	class II
YLR114C	*AVL9*	0.50	yes	class II
YLR200W	*YKE2*	0.25	yes	class II

ORF	standard name	*pet* score	reproducible	class
YLR204W	*QRI5*	0.50	yes	class II
YLR288C	*MEC3*	0.25	yes	class II
YLR369W	*SSQ1*	0.75	yes	class II
YLR377C	*FBP1*	0.75	yes	class II
YLR393W	*ATP10*	0.75	yes	class II
YML088W	*UFO1*	0.50	yes	class II
YMR063W	*RIM9*	0.50	yes	class II
YMR067C	*UBX4*	0.50	yes	class II
YMR071C	*TVP18*	0.50	yes	class II
YMR072W	*ABF2*	1.00	yes	class II
YMR084W		0.50	yes	class II
YMR089C	*YTA12*	1.00	yes	class II
YMR184W	*ADD37*	0.50	yes	class II
YMR207C	*HFA1*	0.50	yes	class II
YNL052W	*COX5A*	1.00	no	class II
YNL133C	*FYV6*	0.25	yes	class II
YNL160W	*YGP1*	0.50	yes	class II
YNL162W	*RPL42A*	0.50	yes	class II
YNL170W		0.75	yes	class II
YNL225C	*CNM67*	0.50	yes	class II
YNR020C	*ATP23*	0.50	yes	class II
YOL008W	*COQ10*	0.75	yes	class II
YOL023W	*IFM1*	0.75	yes	class II
YOL100W	*PKH2*	0.50	yes	class II
YOR026W	*BUB3*	0.25	yes	class II
YOR037W	*CYC2*	0.50	yes	class II
YOR241W	*MET7*	1.00	yes	class II
YOR290C	*SNF2*	0.50	yes	class II
YOR295W	*UAF30*	0.25	yes	class II
YOR305W	*RRG7*	1.00	yes	class II
YPL159C	*PET20*	0.25	yes	class II
YAL047C	*SPC72*	0.50	no	class III
YBL021C	*HAP3*	0.75	yes	class III
YBR018C	*GAL7*	0.00	yes	class III
YBR019C	*GAL10*	0.00	yes	class III
YBR020W	*GAL1*	0.00	yes	class III
YBR036C	*CSG2*	0.25	no	class III
YBR097W	*VPS15*	0.33	yes	class III
YCR053W	*THR4*	0.00	yes	class III
YCR095W-A		0.00	no	class III
YDL041W		0.00	yes	class III
YDL042C	*SIR2*	0.00	yes	class III
YDL090C	*RAM1*	0.00	yes	class III
YDL160C-A	*MHF2*	0.00	yes	class III
YDR005C	*MAF1*	0.00	no	class III
YDR076W	*RAD55*	0.00	no	class III
YDR103W	*STE5*	0.00	yes	class III
YDR126W	*SWF1*	0.00	no	class III
YDR227W	*SIR4*	0.00	yes	class III

ORF	standard name	*pet* score	reproducible	class
YDR269C		0.25	no	class III
YDR270W	*CCC2*	0.50	yes	class III
YDR296W	*MHR1*	0.75	yes	class III
YDR461W	*MFA1*	0.00	yes	class III
YEL004W	*YEA4*	0.00	yes	class III
YEL029C	*BUD16*	0.25	yes	class III
YEL051W	*VMA8*	0.75	yes	class III
YEL072W	*RMD6*	0.00	no	class III
YER052C	*HOM3*	0.00	no	class III
YFL026W	*STE2*	0.00	yes	class III
YGL012W	*ERG4*	0.00	no	class III
YGL156W	*AMS1*	0.00	yes	class III
YGL168W	*HUR1*	0.00	no	class III
YGL206C	*CHC1*	0.50	yes	class III
YGR262C	*BUD32*	0.25	no	class III
YHL007C	*STE20*	0.00	no	class III
YHR005C	*GPA1*	0.00	yes	class III
YHR010W	*RPL27A*	0.00	no	class III
YHR013C	*ARD1*	0.00	no	class III
YHR018C	*ARG4*	0.00	no	class III
YHR030C	*SLT2*	0.00	yes	class III
YHR130C		0.00	no	class III
YHR177W	*ROF1*	0.00	yes	class III
YIL084C	*SDS3*	0.00	yes	class III
YIR009W	*MSL1*	0.00	no	class III
YIR034C	*LYS1*	0.00	no	class III
YJL088W	*ARG3*	0.00	no	class III
YJL094C	*KHA1*	0.00	no	class III
YJL095W	*BCK1*	0.00	yes	class III
YJR005C-A	*LSO1*	0.00	yes	class III
YJR040W	*GEF1*	0.50	no	class III
YJR086W	*STE18*	0.00	yes	class III
YKL135C	*APL2*	0.00	yes	class III
YLR023C	*IZH3*	0.00	no	class III
YLR027C	*AAT2*	0.00	yes	class III
YLR091W	*GEP5*	0.75	yes	class III
YLR244C	*MAP1*	0.00	no	class III
YLR308W	*CDA2*	0.00	yes	class III
YLR320W	*MMS22*	0.00	no	class III
YLR362W	*STE11*	0.00	yes	class III
YLR418C	*CDC73*	0.00	yes	class III
YLR439W	*MRPL4*	0.75	no	class III
YLR442C	*SIR3*	0.00	yes	class III
YML008C	*ERG6*	0.00	no	class III
YML022W	*APT1*	0.00	no	class III
YMR001C-A		0.00	no	class III
YMR058W	*FET3*	0.50	no	class III
YMR097C	*MTG1*	0.75	yes	class III
YMR158W	*MRPS8*	0.75	yes	class III

ORF	standard name	*pet* score	reproducible	class
YMR287C	*DSS1*	0.75	yes	class III
YNL080C	*EOS1*	0.25	yes	class III
YNR048W		0.00	yes	class III
YNR068C		0.00	no	class III
YOL058W	*ARG1*	0.00	no	class III
YOL093W	*TRM10*	0.00	no	class III
YOL162W		0.00	no	class III
YOR033C	*EXO1*	0.00	yes	class III
YOR212W	*STE4*	0.00	yes	class III
YOR371C	*GPB1*	0.00	yes	class III
YPL006W	*NCR1*	0.00	yes	class III
YPL031C	*PHO85*	0.50	no	class III
YPL045W	*VPS16*	0.75	no	class III
YPL049C	*DIG1*	0.00	yes	class III
YPL248C	*GAL4*	0.00	no	class III
YPL254W	*HFI1*	0.50	yes	class III
YPL270W	*MDL2*	0.00	no	class III
YPR021C	*AGC1*	0.00	no	class III

Table S4. Genomic complementation of class II pet mutants by crossing with Δ*mip1*. This table contains the systematic gene name according to SGD, the standard gene name according to SGD, the *pet* score, and the rescue of the *pet* phenotype in heterozygous diploid strains.

Stenger et al. - Table S4

ORF	standard name	*pet* score	Δ*mip1* cross
YGR174C	*CBP4*	0.75	rescued
YHR050W-A		0.33	rescued
YLR393W	*ATP10*	0.75	rescued
YAL048C	*GEM1*	0.50	not rescued
YBL002W	*HTB2*	0.50	not rescued
YBL044W		0.50	not rescued
YBR122C	*MRPL36*	1.00	not rescued
YCR004C	*YCP4*	0.50	not rescued
YCR046C	*IMG1*	1.00	not rescued
YDL040C	*NAT1*	0.25	not rescued
YDL063C	*SYO1*	0.50	not rescued
YDL133C-A	*RPL41B*	0.25	not rescued
YDL146W	*LDB17*	0.50	not rescued
YDR042C		0.50	not rescued
YDR078C	*SHU2*	0.50	not rescued
YDR507C	*GIN4*	0.50	not rescued
YDR518W	*EUG1*	0.50	not rescued
YDR521W		0.25	not rescued
YDR523C	*SPS1*	0.50	not rescued
YEL036C	*ANP1*	0.25	not rescued
YER103W	*SSA4*	0.50	not rescued
YER110C	*KAP123*	0.50	not rescued
YER122C	*GLO3*	0.50	not rescued
YER169W	*RPH1*	0.50	not rescued
YGL107C	*RMD9*	0.75	not rescued
YGL220W	*BOL2*	0.50	not rescued
YGL240W	*DOC1*	0.75	not rescued
YHR120W	*MSH1*	1.00	not rescued
YHR175W-A		0.33	not rescued
YIL012W		0.25	not rescued
YIL041W	*GVP36*	0.25	not rescued
YJL027C		0.25	not rescued
YJR004C	*SAG1*	0.50	not rescued
YJR150C	*DAN1*	0.25	not rescued
YLL006W	*MMM1*	0.50	not rescued
YLR114C	*AVL9*	0.50	not rescued
YLR204W	*QRI5*	0.50	not rescued
YLR288C	*MEC3*	0.25	not rescued
YLR369W	*SSQ1*	0.75	not rescued
YML088W	*UFO1*	0.50	not rescued
YMR071C	*TVP18*	0.50	not rescued
YMR072W	*ABF2*	1.00	not rescued
YMR084W		0.50	not rescued
YMR184W	*ADD37*	0.50	not rescued
YNL160W	*YGP1*	0.50	not rescued
YNL170W		0.75	not rescued
YNL225C	*CNM67*	0.50	not rescued
YOL100W	*PKH2*	0.50	not rescued

**ORF**	**standard name**	***pet* score**	**Δ*mip1* cross**
YOR241W	*MET7*	1.00	not rescued
YOR295W	*UAF30*	0.25	not rescued
YOR305W	*RRG7*	1.00	not rescued

Table S5. Genes required for maintenance of [*ARG8*^{m}] mtDNA and expression of Arg8^{m} in mitochondria. This table contains the systematic gene name according to SGD, the standard gene name according to SGD, the *pet* score, the results of the *ARG8*^{m} screen, and for dubious ORFs a list of overlapping genomic features according to SGD. The table indicates how many independent biological replicates could be analyzed for each mutant and whether these behaved identically. 22 mutants that are either defective in arginine biosynthesis or mating or carry deletions of dubious ORFs are listed at the end of the table. They were excluded from further analysis.

Stenger et al. - Table S5

ORF	standard name	*pet* score	replicates	screen result	reproduced	overlapping ORFs
**mtDNA metabolism**						
YCR028C-A	*RIM1*	1.00	2	no rescue	yes	
YJR144W	*MGM101*	1.00	2	no rescue	yes	
YOR330C	*MIP1*	1.00	2	no rescue	no	
**Mitochondrial transcription**						
YFL036W	*RPO41*	0.75	2	no rescue	yes	
YMR228W	*MTF1*	1.00	2	no rescue	yes	
**Mitochondrial translation and RNA maturation**						
YBL038W	*MRPL16*	0.75	2	ambiguous	yes	
YBL080C	*PET112*	0.75	2	rescue	yes	
YBL090W	*MRP21*	1.00	2	no rescue	yes	
YBR251W	*MRPS5*	0.75	2	rescue	yes	
YBR268W	*MRPL37*	0.75	2	ambiguous	yes	
YBR282W	*MRPL27*	1.00	2	ambiguous	yes	
YCR003W	*MRPL32*	1.00	2	rescue	yes	
YCR024C	*SLM5*	1.00	2	no rescue	yes	
YCR046C	*IMG1*	1.00	2	no rescue	yes	
YCR071C	*IMG2*	1.00	2	rescue	yes	
YDL202W	*MRPL11*	0.75	2	no rescue	yes	
YDR175C	*RSM24*	1.00	2	no rescue	yes	
YDR268W	*MSW1*	0.75	2	no rescue	yes	
YDR296W	*MHR1*	0.75	2	rescue	yes	
YDR322W	*MRPL35*	0.50	2	ambiguous	no	
YDR350C	*ATP22*	0.75	2	ambiguous	yes	
YDR405W	*MRP20*	0.75	2	ambiguous	no	
YEL050C	*RML2*	0.75	2	ambiguous	yes	
YER050C	*RSM18*	1.00	2	no rescue	yes	
YER087W	*AIM10*	1.00	2	rescue	yes	
YGL107C	*RMD9*	0.75	1	no rescue	nd	
YGL129C	*RSM23*	1.00	2	no rescue	yes	
YGL143C	*MRF1*	1.00	1	rescue	nd	
YGR150C	*CCM1*	1.00	2	ambiguous	no	
YGR165W	*MRPS35*	0.50	2	no rescue	yes	
YGR215W	*RSM27*	1.00	2	no rescue	yes	
YGR220C	*MRPL9*	1.00	2	rescue	yes	
YHL004W	*MRP4*	1.00	2	no rescue	yes	
YHR011W	*DIA4*	1.00	2	no rescue	no	
YHR091C	*MSR1*	1.00	2	no rescue	no	
YHR147C	*MRPL6*	1.00	2	rescue	yes	
YJL023C	*PET130*	0.50	1	no rescue	nd	
YJL096W	*MRPL49*	1.00	2	ambiguous	yes	
YJR113C	*RSM7*	1.00	2	rescue	yes	
YKL003C	*MRP17*	1.00	1	ambiguous	nd	
YKL155C	*RSM22*	1.00	2	no rescue	no	
YKL170W	*MRPL38*	1.00	1	rescue	nd	
YKL194C	*MST1*	1.00	2	no rescue	yes	
YKR006C	*MRPL13*	1.00	2	rescue	no	
YKR085C	*MRPL20*	0.75	1	no rescue	nd	

ORF	standard name	*pet* score	replicates	screen result	reproduced	overlapping ORFs
YLR139C	*SLS1*	1.00	2	ambiguous	no	
YLR204W	*QRI5*	0.50	1	rescue	nd	
YMR097C	*MTG1*	0.75	2	rescue	yes	
YMR257C	*PET111*	1.00	2	rescue	yes	
YMR286W	*MRPL33*	1.00	2	no rescue	no	
YMR293C	*HER2*	1.00	2	rescue	yes	
YNL005C	*MRP7*	1.00	2	no rescue	no	
YNL073W	*MSK1*	1.00	2	no rescue	yes	
YNL081C	*SWS2*	1.00	2	no rescue	yes	
YNL177C	*MRPL22*	1.00	2	rescue	yes	
YNL252C	*MRPL17*	0.75	2	no rescue	yes	
YNL284C	*MRPL10*	0.75	1	no rescue	nd	
YNR036C	*MRPS12*	0.75	1	no rescue	nd	
YNR037C	*RSM19*	1.00	2	no rescue	yes	
YOL023W	*IFM1*	0.75	2	rescue	no	
YOL033W	*MSE1*	1.00	2	rescue	no	
YOR150W	*MRPL23*	1.00	2	ambiguous	no	
YOR158W	*PET123*	1.00	2	no rescue	yes	
YOR187W	*TUF1*	1.00	2	no rescue	yes	
YOR201C	*MRM1*	0.50	2	rescue	yes	
YOR205C	*GEP3*	0.75	2	rescue	yes	
YPL005W	*AEP3*	0.75	2	no rescue	yes	
YPL013C	*MRPS16*	1.00	1	no rescue	nd	
YPL097W	*MSY1*	1.00	2	no rescue	yes	
YPL118W	*MRP51*	0.75	2	no rescue	yes	
YPL173W	*MRPL40*	1.00	2	no rescue	no	
YPL183W-A	*RTC6*	0.50	2	no rescue	no	
YPR047W	*MSF1*	0.75	2	rescue	yes	
YPR100W	*MRPL51*	0.75	2	no rescue	no	
YPR116W	*RRG8*	1.00	1	no rescue	nd	
**Respiratory chain components and assembly factors**						
YDR178W	*SDH4*	0.25	2	rescue	yes	
YDR377W	*ATP17*	1.00	1	rescue	nd	
YJL166W	*QCR8*	0.75	2	no rescue	no	
YKL016C	*ATP7*	1.00	1	rescue	nd	
YNL052W	*COX5A*	1.00	2	no rescue	no	
YPL271W	*ATP15*	1.00	1	rescue	nd	
**Mitochondrial fusion**						
YBR179C	*FZO1*	0.75	2	no rescue	yes	
YDR470C	*UGO1*	0.25	2	no rescue	yes	
YGR101W	*PCP1*	0.50	2	no rescue	yes	
YOR211C	*MGM1*	1.00	2	no rescue	no	
**Other mitochondrial function**						
YBL022C	*PIM1*	0.50	1	rescue	nd	
YBR084W	*MIS1*	0.00	1	rescue	nd	
YDL198C	*GGC1*	0.75	2	rescue	no	
YER057C	*HMF1*	0.00	2	no rescue	no	
YER154W	*OXA1*	1.00	2	rescue	yes	
YFL016C	*MDJ1*	1.00	2	no rescue	yes	

ORF	standard name	*pet* score	replicates	screen result	reproduced	overlapping ORFs
YGR257C	*MTM1*	0.50	2	rescue	yes	
YJL200C	*ACO2*	0.00	1	rescue	nd	
YKL134C	*OCT1*	1.00	2	no rescue	yes	
YMR267W	*PPA2*	1.00	2	ambiguous	no	
YOR045W	*TOM6*	0.00	2	rescue	no	
YPL029W	*SUV3*	0.75	2	rescue	yes	
YPL059W	*GRX5*	0.75	1	rescue	nd	
YPL188W	*POS5*	0.25	1	rescue	nd	
YPL270W	*MDL2*	0.00	2	rescue	yes	
**Vacuole-related function**						
YCL005W-A	*VMA9*	1.00	1	rescue	nd	
YDR069C	*DOA4*	0.25	1	rescue	nd	
YDR136C	*VPS61*	0.00	2	rescue	no	
YEL027W	*VMA3*	0.75	1	rescue	nd	
YEL051W	*VMA8*	0.75	2	ambiguous	no	
YGL095C	*VPS45*	0.50	2	ambiguous	yes	
YGR105W	*VMA21*	1.00	2	rescue	yes	
YHR026W	*VMA16*	0.75	1	rescue	nd	
YKL002W	*DID4*	0.75	1	rescue	nd	
YKL041W	*VPS24*	0.00	2	rescue	yes	
YKL080W	*VMA5*	1.00	1	rescue	nd	
YLR261C	*VPS63*	0.00	1	rescue	nd	
YLR396C	*VPS33*	0.75	2	no rescue	yes	
YLR417W	*VPS36*	0.00	2	ambiguous	yes	
YOR106W	*VAM3*	0.00	2	rescue	yes	
YPL002C	*SNF8*	0.00	2	rescue	yes	
YPL084W	*BRO1*	0.00	1	rescue	nd	
**Other function**						
YBR019C	*GAL10*	0.00	2	rescue	yes	
YBR020W	*GAL1*	0.00	1	no rescue	nd	
YBR191W	*RPL21A*	0.00	2	ambiguous	no	
YBR270C	*BIT2*	0.00	2	rescue	no	
YDR005C	*MAF1*	0.00	2	rescue	yes	
YDR017C	*KCS1*	0.00	2	rescue	no	
YDR079C-A	*TFB5*	0.33	2	rescue	no	
YDR123C	*INO2*	0.00	2	rescue	no	
YDR138W	*HPR1*	0.25	2	no rescue	yes	
YDR244W	*PEX5*	0.00	2	rescue	yes	
YDR289C	*RTT103*	0.00	1	rescue	nd	
YDR349C	*YPS7*	0.00	2	rescue	no	
YDR477W	*SNF1*	0.75	1	rescue	nd	
YEL044W	*IES6*	0.25	1	no rescue	nd	
YEL062W	*NPR2*	0.00	1	rescue	nd	
YER070W	*RNR1*	0.50	1	ambiguous	nd	
YFL053W	*DAK2*	0.00	2	rescue	no	
YGL023C	*PIB2*	0.00	2	rescue	no	
YGL084C	*GUP1*	0.25	2	no rescue	yes	
YGL203C	*KEX1*	0.00	2	rescue	no	
YGR077C	*PEX8*	0.00	2	rescue	no	

ORF	standard name	*pet* score	replicates	screen result	reproduced	overlapping ORFs
YHL011C	*PRS3*	0.00	2	no rescue	yes	
YJL121C	*RPE1*	0.50	1	rescue	nd	
YJL157C	*FAR1*	0.00	1	no rescue	nd	
YJR073C	*OPI3*	0.00	2	rescue	yes	
YKL038W	*RGT1*	0.00	1	ambiguous	nd	
YKL179C	*COY1*	0.00	2	rescue	no	
YKR055W	*RHO4*	0.00	1	rescue	nd	
YLR182W	*SWI6*	0.00	2	no rescue	no	
YLR350W	*ORM2*	0.25	2	no rescue	no	
YLR351C	*NIT3*	0.00	2	no rescue	no	
YLR403W	*SFP1*	0.00	1	rescue	nd	
YMR180C	*CTL1*	0.00	2	rescue	no	
YMR285C	*NGL2*	0.00	2	rescue	no	
YNL064C	*YDJ1*	0.25	2	no rescue	no	
YNL286W	*CUS2*	0.00	2	rescue	no	
YNL323W	*LEM3*	0.00	1	rescue	nd	
YNL327W	*EGT2*	0.00	2	rescue	no	
YOL039W	*RPP2A*	0.00	2	no rescue	no	
YOL081W	*IRA2*	0.00	1	rescue	nd	
YOL108C	*INO4*	0.00	1	rescue	nd	
YOL145C	*CTR9*	0.00	1	rescue	nd	
YOR014W	*RTS1*	0.00	1	rescue	nd	
YOR021C	*SFM1*	0.00	2	rescue	no	
YOR027W	*STI1*	0.00	2	no rescue	no	
YOR171C	*LCB4*	0.00	2	rescue	no	
YOR295W	*UAF30*	0.25	1	no rescue	nd	
YOR307C	*SLY41*	0.00	2	rescue	no	
YPL031C	*PHO85*	0.50	1	ambiguous	nd	
YPL069C	*BTS1*	0.00	2	ambiguous	no	
**Unknown function**						
YDR065W	*RRG1*	1.00	2	rescue	yes	
YDR114C		1.00	2	rescue	no	*MRX14*
YLR255C		0.00	2	no rescue	no	
YMR066W	*SOV1*	0.75	2	no rescue	yes	
YNL133C	*FYV6*	0.25	2	rescue	yes	
YNL184C		1.00	1	no rescue	nd	*MRPL19*
YNL213C	*RRG9*	1.00	1	rescue	nd	
YPL056C	*LCL1*	0.00	1	no rescue	nd	
YPL205C		0.00	2	rescue	no	
**Arginine biosynthesis**						
YER069W	*ARG5,6*	0.00	2	rescue	yes	
YHR018C	*ARG4*	0.00	2	rescue	yes	
YJL088W	*ARG3*	0.00	2	rescue	yes	
YJR109C	*CPA2*	0.00	2	rescue	yes	
YOL058W	*ARG1*	0.00	2	rescue	yes	
YOR303W	*CPA1*	0.00	2	rescue	yes	
**Dubious ORF**						
YCL007C		1.00	2	rescue	no	*VMA9*
YER068C-A		0.00	2	rescue	yes	*ARG5,6*

ORF	standard name	*pet* score	replicates	screen result	reproduced	overlapping ORFs
YGL218W		0.75	2	rescue	no	*MDM34*
YGR219W		0.75	2	rescue	yes	*MRPL9*
YJL120W		0.75	2	ambiguous	no	*RPE1*
YJL175W		0.25	1	no rescue	nd	*SWI3*
YJR114W		1.00	2	no rescue	yes	*RSM7*
YKL076C	*PSY1*	0.00	1	no rescue	nd	*YKL075C*
YKL169C		1.00	2	rescue	yes	*MRPL38*
YKL177W		0.00	2	no rescue	yes	*STE3*
**Mating**						
YCR020W-B	*HTL1*	0.25	1	no rescue	nd	
YGL032C	*AGA2*	0.00	2	rescue	no	
YHL007C	*STE20*	0.00	1	no rescue	nd	
YKL178C	*STE3*	0.00	2	no rescue	yes	
YLR452C	*SST2*	0.00	2	no rescue	no	
YML008C	*ERG6*	0.00	2	no rescue	yes	

Table S6. Genes required for maintenance of [*ARG8*^{m}] mtDNA. 89 strains that showed an instable mtDNA phenotype (see Table S5) were re-analyzed by cytoduction with the [*ARG8*^{m} *rho*$^{+}$] mitochondrial genome and mating with the Δ*arg8* [*rho*^{0}] strain. Then, cells were stained with DAPI and analyzed by fluorescence microscopy. nd, not determined.

Stenger et al. - Table S6

ORF	standard name	growth on media lacking arginine		DAPI staining:
		after cytoduction	after mating with Δ*arg8* [*rho* ^{0}]	mtDNA
YPL005W	*AEP3*	no	no	no
YDR350C	*ATP22*	no	no	no
YPL069C	*BTS1*	no	yes	yes
YGR150C	*CCM1*	nd	nd	nd
YNL052W	*COX5A*	no	no	no
YHR011W	*DIA4*	no	no	no
YJL157C	*FAR1*	no	yes	yes
YBR179C	*FZO1*	no	no	no
YBR020W	*GAL1*	no	yes	yes
YGL084C	*GUP1*	yes	yes	nd
YER057C	*HMF1*	no	yes	yes
YDR138W	*HPR1*	no	no	no
YEL044W	*IES6*	yes	yes	nd
YCR046C	*IMG1*	no	no	no
YPL056C	*LCL1*	no	no	no
YFL016C	*MDJ1*	no	no	no
YOR211C	*MGM1*	no	no	no
YJR144W	*MGM101*	no	no	no
YOR330C	*MIP1*	no	no	no
YKL003C	*MRP17*	no	no	no
YDR405W	*MRP20*	no	no	no
YBL090W	*MRP21*	no	no	no
YHL004W	*MRP4*	no	no	no
YPL118W	*MRP51*	no	no	no
YNL005C	*MRP7*	no	no	no
YNL284C	*MRPL10*	no	no	no
YDL202W	*MRPL11*	no	no	no
YBL038W	*MRPL16*	no	no	no
YNL252C	*MRPL17*	no	no	no
YKR085C	*MRPL20*	no	no	no
YOR150W	*MRPL23*	no	no	no
YBR282W	*MRPL27*	no	no	no
YMR286W	*MRPL33*	no	no	yes
YDR322W	*MRPL35*	no	no	no
YBR268W	*MRPL37*	no	no	no
YPL173W	*MRPL40*	no	no	yes
YJL096W	*MRPL49*	no	no	no
YPR100W	*MRPL51*	no	no	no
YNR036C	*MRPS12*	no	no	no
YPL013C	*MRPS16*	no	no	no
YGR165W	*MRPS35*	no	no	no
YNL073W	*MSK1*	no	no	no
YHR091C	*MSR1*	no	no	yes
YKL194C	*MST1*	no	no	no
YDR268W	*MSW1*	no	no	no
YPL097W	*MSY1*	no	no	no
YMR228W	*MTF1*	no	no	no

ORF	standard name	growth on media lacking arginine		DAPI staining:
		after cytoduction	after mating with Δ*arg8* [*rho* ^{0}]	mtDNA
YLR351C	*NIT3*	nd	nd	nd
YKL134C	*OCT1*	no	no	no
YLR350W	*ORM2*	yes	yes	nd
YGR101W	*PCP1*	no	no	no
YOR158W	*PET123*	no	no	no
YJL023C	*PET130*	no	no	no
YPL031C	*PHO85*	nd	nd	nd
YMR267W	*PPA2*	no	no	no
YHL011C	*PRS3*	yes	yes	nd
YJL166W	*QCR8*	no	yes	yes
YKL038W	*RGT1*	yes	yes	nd
YCR028C-A	*RIM1*	no	no	no
YGL107C	*RMD9*	no	no	yes
YEL050C	*RML2*	no	no	no
YER070W	*RNR1*	no	yes	yes
YBR191W	*RPL21A*	no	yes	yes
YFL036W	*RPO41*	no	no	no
YOL039W	*RPP2A*	no	no	no
YPR116W	*RRG8*	no	no	no
YER050C	*RSM18*	no	no	no
YNR037C	*RSM19*	no	no	no
YKL155C	*RSM22*	nd	nd	nd
YGL129C	*RSM23*	no	no	no
YDR175C	*RSM24*	no	no	no
YGR215W	*RSM27*	no	no	no
YPL183W-A	*RTC6*	nd	nd	nd
YCR024C	*SLM5*	nd	nd	nd
YLR139C	*SLS1*	nd	nd	nd
YMR066W	*SOV1*	no	no	no
YOR027W	*STI1*	yes	yes	nd
YLR182W	*SWI6*	nd	nd	nd
YNL081C	*SWS2*	no	no	no
YOR187W	*TUF1*	no	no	yes
YOR295W	*UAF30*	no	no	no
YDR470C	*UGO1*	no	no	no
YEL051W	*VMA8*	no	yes	yes
YLR396C	*VPS33*	no	no	no
YLR417W	*VPS36*	no	yes	yes
YGL095C	*VPS45*	no	yes	yes
YNL064C	*YDJ1*	no	yes	yes
YLR255C	*YLR255C*	yes	yes	nd
YNL184C	*YNL184C*	nd	nd	nd

Table S7. Combined results of five systematic screens to identify genes required for maintenance of mtDNA. The column "this study" lists the 57 [*rho*^{0}] mutants shown in Table 4; Merz and Westermann (2009) identified 119 mutants lacking mtDNA in the yeast deletion collection after mating with Δ*mip1* and cytoduction with wild type mtDNA [18]; Zhang and Singh (2014) identified 102 [*rho*^{0}] strains by DAPI staining of 466 *pet* mutants [71]; Göke et al. (2020) identified 180 mutants lacking mtDNA by colony hybridization of the yeast deletion collection with probes specific for mtDNA [72]; and Puddu et al. (2019) identified 303 biological replicates comprising 165 gene deletions with mtDNA copy number of less than 1 by genome sequencing of strains of the yeast deletion collection [73]. It should be noted that the screens performed by Merz and Westermann (2009) [18], Göke et al. (2020) [72], and Puddu et al. (2019) [73] cannot discriminate between [*rho*^{0}] and [*rho*$^-$] strains.

References

18. Merz S, Westermann B (**2009**). Genome-wide deletion mutant analysis reveals genes required for respiratory growth, mitochondrial genome maintenance and mitochondrial protein synthesis in *Saccharomyces cerevisiae*. **Genome Biol** 10(9): R95. doi: 10.1186/gb-2009-10-9-r95.

71. Zhang H, Singh KK (**2014**). Global genetic determinants of mitochondrial DNA copy number. **PLoS One** 9(8): e105242. doi: 10.1371/journal.pone.0105242.

72. Göke A, Schrott S, Mizrak A, Belyy V, Osman C, Walter P (**2020**). Mrx6 regulates mitochondrial DNA copy number in *Saccharomyces cerevisiae* by engaging the evolutionarily conserved Lon protease Pim1. **Mol Biol Cell** 31(7): 527-545. doi: 10.1091/mbc.E19-08-0470.

73. Puddu F, Herzog M, Selivanova A, Wang S, Zhu J, Klein-Lavi S, Gordon M, Meirman R, Millan-Zambrano G, Ayestaran I, Salguero I, Sharan R, Li R, Kupiec M, Jackson SP (**2019**). Genome architecture and stability in the *Saccharomyces cerevisiae* knockout collection. **Nature** 573(7774): 416-420. doi: 10.1038/s41586-019-1549-9.

Stenger et al. - Table S7

Standard name	ORF	*pet* score	wt mtDNA present				
			this study	ref. [18]	ref. [71]	ref. [72]	ref. [73]
**Lack of mtDNA reported in all five screens**							
DIA4	*YHR011W*	1.00	no	no	no	no	no
FZO1	*YBR179C*	0.75	no	no	no	no	no
MDJ1	*YFL016C*	1.00	no	no	no	no	no
MGM101	*YJR144W*	1.00	no	no	no	no	no
MIP1	*YOR330C*	1.00	no	no	no	no	no
MRP21	*YBL090W*	1.00	no	no	no	no	no
MRPL17	*YNL252C*	0.75	no	no	no	no	no
MRPL23	*YOR150W*	1.00	no	no	no	no	no
MRPL27	*YBR282W*	1.00	no	no	no	no	no
MRPL37	*YBR268W*	0.75	no	no	no	no	no
MRPS16	*YPL013C*	1.00	no	no	no	no	no
MSY1	*YPL097W*	1.00	no	no	no	no	no
OCT1	*YKL134C*	1.00	no	no	no	no	no
RML2	*YEL050C*	0.75	no	no	no	no	no
RRG8	*YPR116W*	1.00	no	no	no	no	no
RSM18	*YER050C*	1.00	no	no	no	no	no
RSM19	*YNR037C*	1.00	no	no	no	no	no
RSM23	*YGL129C*	1.00	no	no	no	no	no
RSM27	*YGR215W*	1.00	no	no	no	no	no
SWS2	*YNL081C*	1.00	no	no	no	no	no
**Lack of mtDNA reported in four screens**							
AEP3	*YPL005W*	0.75	no		no	no	no
CCM1	*YGR150C*	1.00		no	no	no	no
MEF2	*YJL102W*	0.75		no	no	no	no
MET7	*YOR241W*	1.00		no	no	no	no
MGM1	*YOR211C*	1.00	no	no		no	no
MRF1	*YGL143C*	1.00		no	no	no	no
MRP20	*YDR405W*	0.75	no		no	no	no
MRP51	*YPL118W*	0.75	no		no	no	no
MRP7	*YNL005C*	1.00	no		no	no	no
MRPL11	*YDL202W*	0.75	no		no	no	no
MRPL16	*YBL038W*	0.75	no		no	no	no
MRPL32	*YCR003W*	1.00		no	no	no	no
MRPL35	*YDR322W*	0.50	no		no	no	no
MRPL38	*YKL170W*	1.00		no	no	no	no
MRPL40	*YPL173W*	1.00		no	no	no	no
MRPL49	*YJL096W*	1.00	no	no		no	no
MRPL51	*YPR100W*	0.75	no		no	no	no
MRPL6	*YHR147C*	1.00		no	no	no	no
MRPL8	*YJL063C*	0.75		no	no	no	no
MRPS12	*YNR036C*	0.75	no		no	no	no
MRPS8	*YMR158W*	0.75		no	no	no	no
MSE1	*YOL033W*	1.00		no	no	no	no
MSH1	*YHR120W*	1.00		no	no	no	no
MSK1	*YNL073W*	1.00	no	no	no	no	
MSW1	*YDR268W*	0.75	no		no	no	no

Standard name	ORF	*pet* score	wt mtDNA present				
			this study	ref. [18]	ref. [71]	ref. [72]	ref. [73]
MTF1	*YMR228W*	1.00	no	no		no	no
MTG2	*YHR168W*	0.75		no	no	no	no
NAM2	*YLR382C*	0.75		no	no	no	no
PET100	*YDR079W*	1.00		no	no	no	no
PET123	*YOR158W*	1.00	no	no		no	no
PPA2	*YMR267W*	1.00	no	no		no	no
RSM22	*YKL155C*	1.00		no	no	no	no
RSM24	*YDR175C*	1.00	no	no		no	no
SLM5	*YCR024C*	1.00		no	no	no	no
SOV1	*YMR066W*	0.75	no		no	no	no
YTA12	*YMR089C*	1.00		no	no	no	no
**Lack of mtDNA reported in three screens**							
ABF2	*YMR072W*	1.00			no	no	no
AFG3	*YER017C*	0.75			no	no	no
ATP15	*YPL271W*	1.00		no		no	no
ATP22	*YDR350C*	0.75	no	no		no	
ATP25	*YMR098C*	1.00		no		no	no
ATP4	*YPL078C*	1.00		no	no	no	
ATP5	*YDR298C*	1.00			no	no	no
DOC1	*YGL240W*	0.75		no		no	no
DSS1	*YMR287C*	0.75		no		no	no
EUG1	*YDR518W*	0.50			no	no	no
GEP5	*YLR091W*	0.75		no	no	no	
GTF1	*YGR102C*	0.50		no	no		no
HER2	*YMR293C*	1.00		no		no	no
HMI1	*YOL095C*	0.75		no	no		no
IMG1	*YCR046C*	1.00	no			no	no
IMG2	*YCR071C*	1.00		no		no	no
ISA2	*YPR067W*	1.00		no	no	no	
MEF1	*YLR069C*	0.75		no	no	no	
MHR1	*YDR296W*	0.75		no		no	no
MRH4	*YGL064C*	0.25			no	no	no
MRM1	*YOR201C*	0.50			no	no	no
MRP1	*YDR347W*	0.75		no		no	no
MRP10	*YDL045W-A*	0.75		no		no	no
MRP17	*YKL003C*	1.00	no	no		no	
MRPL10	*YNL284C*	0.75	no			no	no
MRPL13	*YKR006C*	1.00		no		no	no
MRPL20	*YKR085C*	0.75	no		no		no
MRPL22	*YNL177C*	1.00		no		no	no
MRPL24	*YMR193W*	0.75		no	no		no
MRPL25	*YGR076C*	0.75		no	no		no
MRPL7	*YDR237W*	0.75			no	no	no
MRPL9	*YGR220C*	1.00			no	no	no
MRPS28	*YDR337W*	0.75		no	no		no
MRPS35	*YGR165W*	0.50	no			no	no
MRPS5	*YBR251W*	0.75		no	no	no	
MSD1	*YPL104W*	0.75		no	no		no

Standard name	ORF	*pet* score	wt mtDNA present				
			this study	ref. [18]	ref. [71]	ref. [72]	ref. [73]
MSF1	*YPR047W*	0.75			no	no	no
MSR1	*YHR091C*	1.00		no	no	no	
MSS116	*YDR194C*	0.75		no		no	no
MST1	*YKL194C*	1.00	no	no		no	
MTF2	*YDL044C*	0.75		no		no	no
MTM1	*YGR257C*	0.50			no	no	no
PCP1	*YGR101W*	0.50	no			no	no
PET130	*YJL023C*	0.50	no			no	no
RIM1	*YCR028C-A*	1.00	no			no	no
RPO41	*YFL036W*	0.75	no		no	no	
RRF1	*YHR038W*	0.75		no	no		no
RRG1	*YDR065W*	1.00		no	no	no	
RRG7	*YOR305W*	1.00			no	no	no
RSM7	*YJR113C*	1.00			no	no	no
SLS1	*YLR139C*	1.00		no		no	no
SSQ1	*YLR369W*	0.75		no	no	no	
SUV3	*YPL029W*	0.75			no	no	no
TUF1	*YOR187W*	1.00		no	no	no	
YDR114C	*YDR114C*	1.00		no	no	no	
YKL169C	*YKL169C*	1.00		no		no	no
YNL184C	*YNL184C*	1.00		no		no	no
YOR199W	*YOR199W*	0.50			no	no	no
**Lack of mtDNA reported in two screens**							
AEP1	*YMR064W*	1.00		no		no	
AEP2	*YMR282C*	0.75		no			no
ATP14	*YLR295C*	0.50		no	no		
ATP17	*YDR377W*	1.00		no		no	
ATP7	*YKL016C*	1.00				no	no
ERG5	*YMR015C*	0.25		no			no
EXO5	*YBR163W*	0.50				no	no
GEP3	*YOR205C*	0.75			no	no	
GGC1	*YDL198C*	0.75				no	no
GIN4	*YDR507C*	0.50				no	no
GLO3	*YER122C*	0.50				no	no
GRX5	*YPL059W*	0.75			no	no	
HEM14	*YER014W*	0.67			no	no	
HPR1	*YDR138W*	0.25	no				no
IBA57	*YJR122W*	1.00			no	no	
IRC19	*YLL033W*	0.75		no			no
ISA1	*YLL027W*	1.00		no		no	
ISM1	*YPL040C*	0.25			no		no
KAP123	*YER110C*	0.50			no	no	
LCB5	*YLR260W*	0.75			no	no	
MDM12	*YOL009C*	0.50		no			no
MMM1	*YLL006W*	0.50				no	no
MRP2	*YPR166C*	0.50			no		no
MRP4	*YHL004W*	1.00	no			no	
MRPL15	*YLR312W-A*	1.00		no		no	

Standard name	ORF	*pet* score	wt mtDNA present				
			this study	ref. [18]	ref. [71]	ref. [72]	ref. [73]
MRPL31	*YKL138C*	0.75		no	no		
MRPL33	*YMR286W*	1.00				no	no
MRPL4	*YLR439W*	0.75		no			no
MRPS17	*YMR188C*	0.50		no			no
MRX14	*YDR115W*	0.75		no	no		
MSM1	*YGR171C*	0.75		no			no
MTG1	*YMR097C*	0.75			no	no	
OXA1	*YER154W*	1.00		no		no	
PET112	*YBL080C*	0.75		no			no
PET309	*YLR067C*	1.00		no			no
PHO85	*YPL031C*	0.50				no	no
PIF1	*YML061C*	0.75		no			no
PPT2	*YPL148C*	0.75		no	no		
QRI5	*YLR204W*	0.50			no	no	
RMD9	*YGL107C*	0.75				no	no
RNR4	*YGR180C*	1.00			no	no	
RPL1B	*YGL135W*	0.75			no		no
RRG9	*YNL213C*	1.00		no		no	
RTC6	*YPL183W-A*	0.50				no	no
UGO1	*YDR470C*	0.25	no			no	
VPS33	*YLR396C*	0.75	no			no	
VPS45	*YGL095C*	0.50			no	no	
YGR219W	*YGR219W*	0.75				no	no
YOR200W	*YOR200W*	1.00				no	no
YPR099C	*YPR099C*	0.75				no	no
**Lack of mtDNA reported in one screen**							
ACO1	*YLR304C*	0.75		no			
ADD37	*YMR184W*	0.50				no	
AGP2	*YBR132C*	0.00				no	
AIM10	*YER087W*	1.00				no	
AIM22	*YJL046W*	1.00					no
ANP1	*YEL036C*	0.25				no	
APN1	*YKL114C*	0.25		no			
APN2	*YBL019W*	0.25		no			
ATG34	*YOL083W*	0.25		no			
ATP10	*YLR393W*	0.75					no
ATP3	*YBR039W*	1.00		no			
AVL9	*YLR114C*	0.50				no	
BOL2	*YGL220W*	0.50				no	
BRE1	*YDL074C*	0.25					no
BRE2	*YLR015W*	0.00					no
BUD16	*YEL029C*	0.25			no		
CBP2	*YHL038C*	1.00		no			
CBS1	*YDL069C*	0.75					no
CEM1	*YER061C*	1.00					no
CLC1	*YGR167W*	0.50		no			
COX5A	*YNL052W*	1.00	no				
CYC3	*YAL039C*	0.75		no			

Standard name	ORF	*pet* score	wt mtDNA present				
			this study	ref. [18]	ref. [71]	ref. [72]	ref. [73]
DCS1	*YLR270W*	0.50				no	
DID4	*YKL002W*	0.75					no
ELO3	*YLR372W*	0.00					no
FAB1	*YFR019W*	0.25					no
GDH1	*YOR375C*	0.25		no			
GEM1	*YAL048C*	0.50				no	
GET1	*YGL020C*	0.00					no
HAP3	*YBL021C*	0.75		no			
HDA2	*YDR295C*	0.25				no	
HFI1	*YPL254W*	0.50		no			
HSP31	*YDR533C*	0.25					no
HTB2	*YBL002W*	0.50				no	
HTD2	*YHR067W*	1.00					no
IFM1	*YOL023W*	0.75					no
INH1	*YDL181W*	0.33				no	
IRC3	*YDR332W*	0.50					no
LCL1	*YPL056C*	0.00	no				
LDB17	*YDL146W*	0.50				no	
MAC1	*YMR021C*	0.75					no
MBP1	*YDL056W*	0.50					no
MDM10	*YAL010C*	0.50					no
MDM38	*YOL027C*	0.25					no
MEC3	*YLR288C*	0.25				no	
MOT2	*YER068W*	0.25					no
MRP49	*YKL167C*	0.25					no
MRPL19	*YNL185C*	1.00		no			
MRPL28	*YDR462W*	0.25					no
MRPL36	*YBR122C*	1.00				no	
MRPS9	*YBR146W*	0.25		no			
MRX1	*YER077C*	0.25					no
NAT1	*YDL040C*	0.25				no	
NRP1	*YDL167C*	0.50				no	
PAF1	*YBR279W*	0.00					no
PEP3	*YLR148W*	0.50				no	
PIM1	*YBL022C*	0.50				no	
PKH2	*YOL100W*	0.50				no	
PRO1	*YDR300C*	0.25				no	
PTC1	*YDL006W*	0.00					no
QRI7	*YDL104C*	0.25			no		
RNR1	*YER070W*	0.50		no			
RPB4	*YJL140W*	0.00				no	
RPH1	*YER169W*	0.50				no	
RPP2A	*YOL039W*	0.00	no				
RSM25	*YIL093C*	0.25					no
RSM26	*YJR101W*	0.00				no	
SHU2	*YDR078C*	0.50				no	
SLA1	*YBL007C*	0.25					no
SOD2	*YHR008C*	0.25					no

Standard name	ORF	*pet* score	wt mtDNA present				
			this study	ref. [18]	ref. [71]	ref. [72]	ref. [73]
SPS1	*YDR523C*	0.50				no	
SRF1	*YDL133W*	0.25		no			
SSA4	*YER103W*	0.50				no	
SUT1	*YGL162W*	0.00					no
SYO1	*YDL063C*	0.50				no	
TDA5	*YLR426W*	0.00					no
THP1	*YOL072W*	0.00					no
TPM1	*YNL079C*	0.00					no
TVP18	*YMR071C*	0.50				no	
UAF30	*YOR295W*	0.25	no				
UFO1	*YML088W*	0.50				no	
VID22	*YLR373C*	0.00					no
VPS41	*YDR080W*	0.00					no
XYL2	*YLR070C*	0.25		no			
YBL012C	*YBL012C*	0.25				no	
YBL044W	*YBL044W*	0.50				no	
YCP4	*YCR004C*	0.50				no	
YDL032W	*YDL032W*	0.50					no
YDL057W	*YDL057W*	0.33				no	
YDL062W	*YDL062W*	0.25				no	
YDL129W	*YDL129W*	0.25		no			
YDR042C	*YDR042C*	0.50				no	
YDR521W	*YDR521W*	0.25				no	
YGL218W	*YGL218W*	0.75					no
YGP1	*YNL160W*	0.50				no	
YHR175W-A	*YHR175W-A*	0.33				no	
YIL014C-A	*YIL014C-A*	0.25					no
YJL022W	*YJL022W*	0.25					no
YJL027C	*YJL027C*	0.25				no	
YJR114W	*YJR114W*	1.00				no	
YLR149C	*YLR149C*	0.25					no
YMC1	*YPR058W*	0.00					no
YMR084W	*YMR084W*	0.50				no	
YNL170W	*YNL170W*	0.75				no	
